# Generation of an Obese Diabetic Mouse Model upon Conditional *Atrx* Disruption

**DOI:** 10.3390/cancers15113018

**Published:** 2023-06-01

**Authors:** Tiago Bordeira Gaspar, Tito Teles Jesus, Maria Teresa Azevedo, Sofia Macedo, Mariana Alves Soares, Rui Sousa Martins, Rúben Leite, Lia Rodrigues, Daniela Ferreira Rodrigues, Luís Cardoso, Inês Borges, Sule Canberk, Fátima Gärtner, Leandro Miranda-Alves, José Manuel Lopes, Paula Soares, João Vinagre

**Affiliations:** 1 Instituto de Investigação e Inovação em Saúde (i3S), University of Porto, 4200-135 Porto, Portugal; tgaspar@ipatimup.pt (T.B.G.); tjesus@ipatimup.pt (T.T.J.); tazevedo@i3s.up.pt (M.T.A.); amacedo@ipatimup.pt (S.M.); mrnlvs37@ufrj.br (M.A.S.); rmartins@i3s.up.pt (R.S.M.); rleite@i3s.up.pt (R.L.); lrodrigues@i3s.up.pt (L.R.); drodrigues@indicalab.com (D.F.R.); lcardoso@i3s.up.pt (L.C.); scanberk@ipatimup.pt (S.C.); fgartner@ipatimup.pt (F.G.); jmlopes@ipatimup.pt (J.M.L.); psoares@ipatimup.pt (P.S.); 2 Institute of Molecular Pathology and Immunology of the University of Porto (IPATIMUP), 4200-135 Porto, Portugal; 3 Institute of Biomedical Sciences Abel Salazar (ICBAS), University of Porto, 4050-313 Porto, Portugal; 4 Faculty of Medicine of the University of Porto (FMUP), 4200-319 Porto, Portugal; 5 Laboratório de Endocrinologia Experimental (LEEx), Instituto de Ciências Biomédicas (ICB), Universidade Federal do Rio de Janeiro, Rio de Janeiro 21941-902, Brazil; leandro.alves@icb.ufrj.br; 6 Programa de Pós-Graduação em Endocrinologia, Faculdade de Medicina, Universidade Federal do Rio de Janeiro, Rio de Janeiro 21941-902, Brazil; 7 Faculty of Sciences of the University of Porto (FCUP), 4169-007 Porto, Portugal; 8 School of Health (ESS), Polytechnic Institute of Porto (IPP), Rua Dr. António Bernardino de Almeida, 4200-072 Porto, Portugal; 9 Institute of Molecular and Cell Biology (IBMC), University of Porto, 4200-135 Porto, Portugal; 10 Department of Endocrinology, Diabetes and Metabolism, Centro Hospitalar e Universitário de Coimbra, 3000-075 Coimbra, Portugal; 11 Centro de Diagnóstico Veterinário (Cedivet), 4200-071 Porto, Portugal; ines.borges@cedivet.pt; 12 Department of Pathology, Centro Hospitalar Universitário de São João (CHUSJ), 4200-319 Porto, Portugal

**Keywords:** Atrx, conditional mouse model, endocrine dysfunction, fatty pancreas, pancreatic fatty replacement, hyperglycaemia, hepatic stetosis, inflammageing, obesity, Pdx1-Cre

## Abstract

**Simple Summary:**

*ATRX* mutations occur in up to 17% of human pancreatic neuroendocrine tumours (PanNETs), and recent evidence points towards its inability to drive PanNET formation in mouse pancreas while predisposing individuals to inflammageing. Aiming to explore the additional non-tumourigenic consequences of *Atrx* deletion, we characterised an aged series of *Atrx* conditional disruption in β cells using the *Pdx1* promoter. Homozygous mice (*P.Atrx^HOM^*) exhibited obesity, diabetes, glucose intolerance, and pancreatic adiposity at a higher extent than age- and sex-matched controls (*P.Atrx^WT^*).

**Abstract:**

Atrx loss was recently ascertained as insufficient to drive pancreatic neuroendocrine tumour (PanNET) formation in mice islets. We have identified a preponderant role of Atrx in the endocrine dysfunction in a *Rip-Cre;Atrx^KO^* genetically engineered mouse model (GEMM). To validate the impact of a different *Cre*-driver line, we used similar methodologies and characterised the *Pdx1-Cre;Atrx^KO^* (*P.Atrx^KO^*) GEMM to search for PanNET formation and endocrine fitness disruption for a period of up to 24 months. Male and female mice presented different phenotypes. Compared to *P.Atrx^WT^*, *P.Atrx^HOM^* males were heavier during the entire study period, hyperglycaemic between 3 and 12 mo., and glucose intolerant only from 6 mo.; in contrast, *P.Atrx^HOM^* females started exhibiting increased weight gains later (after 6 mo.), but diabetes or glucose intolerance was detected by 3 mo. Overall, all studied mice were overweight or obese from early ages, which challenged the histopathological evaluation of the pancreas and liver, especially after 12 mo. Noteworthily, losing *Atrx* predisposed mice to an increase in intrapancreatic fatty infiltration (FI), peripancreatic fat deposition, and macrovesicular steatosis. As expected, no animal developed PanNETs. An obese diabetic GEMM of disrupted *Atrx* is presented as potentially useful for metabolic studies and as a putative candidate for inserting additional tumourigenic genetic events.

## 1. Introduction

The ATRX chromatin remodeller (ATRX) is an ATP-dependent helicase that belongs to the switch 2/sucrose non-fermentable 2 (SWI2/SNF2) family of chromatin remodelling proteins [1,2]. SWI2/SNF2 proteins participate in DNA recombination and repair mechanisms, nucleosome remodelling, and transcriptional regulation, amid other biological processes [2]. ATRX is a nuclear protein with a ubiquitous distribution, with the highest levels reported in the foetal brain implying a relevant role in brain development [3]. ATRX is also highly conserved between mice and humans (85% homology) [2]. The ATRX protein essentially contains three functional and highly conserved domains: (1) an ATPase/helicase C-terminal domain that provides DNA-dependent ATPase activity [4,5]; (2) an ATRX-DNMT3-DNMT3L (ADD) domain at the N-terminal [4,6], which is a plant homeodomain (PHD)-like zinc finger, structurally similar to DNA methyltransferases (DNMTs), that is involved in heterochromatin organisation, comprising three subdomains: a GATA-like, PHD-like, and a C-terminal α helix [4,7,8]; and (3) a centrally located binding domain for the death domain-associated protein (DAXX), essential for H3.3 deposition [9,10]. The multiple functions of ATRX have been recently reviewed in detail by Valenzuela et al. [5]. ATRX plays essential functions toward heterochromatin homeostasis [11] and recruits DAXX, forming a nuclear chaperone complex that facilitates the recruitment, deposition, and integration of the histone variant H3.3 at heterochromatic regions [12,13,14]. This H3.3 deposition ensures that the chromatin and DNA’s B-form conformation remain stable, which prevents the collapse of a stalled replication fork [15]. ATRX also has essential cellular functions independent of DAXX (e.g., genomic stability, regulation of gene expression, and the binding and resolution of quadruplex G- and C-rich repeats (G4 and i-Motifs, respectively) that could compromise genomic stability [4,5,16]. In addition, ATRX binds and regulates regions enriched for H3K9me3 via its ADD domain and HP1α [11].

Importantly, ATRX, DAXX, H3.3, and promyelocytic leukaemia (PML) nuclear bodies (PML-NBs) are responsible for the epigenetic silencing of transposable elements such as the endogenous retroviral elements (ERVs) in the mouse genome [10,17,18], as well as in the defence against viral infection [19,20,21]. It is plausible that the derepression of ERV silencing may occur upon loss of either of these genetic players, activating transposons and leading to the expression of endogenous genes with an impact on carcinogenesis [18].

In recent years, as we have witnessed the emergence of high-throughput sequencing techniques, *ATRX* and *DAXX* mutations have been reported in pancreatic neuroendocrine tumours (PanNETs) (10% and 20%, respectively) [22,23,24,25]. The prevalence of these mutations stresses their importance as putative tumour driver events. A correlation between mutations and the telomerase-independent mechanism of the alternative lengthening of telomeres (ALT) was also established [15,26], with particular importance to PanNETs [27,28,29] that rely on this telomere maintenance mechanism (TMM) twice as often (30%) as other overall tumour types (10–15%, on average) [30]. Losing *ATRX* or *DAXX* in addition to ALT positivity correlates with chromosome instability, higher tumour grading, and unfavourable prognosis in PanNET patients [27,28,29,31,32].

Genetically engineered mouse models (GEMMs) are the most powerful tool to study the PanNETs’ multistep tumourigenic pathway, regarding tumour initiation and progression, under an immune-competent phenotype [33]. Despite the increasing acceptance of the chromatin remodellers’ utility as prognostic markers in the context of human PanNETs, their specific contribution to PanNET tumourigenesis is still underexplored. The first GEMMs tackling the pancreas-specific functions of chromatin remodellers have appeared recently in the literature. *Atrx* and *Daxx* could not drive neuroendocrine tumour progression nor intensify the effect of *Pten* or *Men1* homozygous deletion [18,34,35]. However, *Atrx* was relevant to the tumourigenesis of the exocrine pancreas, as the *Mist1-Cre;Kras^HOM^;Atrx^HOM^* (*M.K.Atrx^HOM^*) double-mutant mice developed precursor lesions sooner than if only losing *Kras* [36]. *Atrx* and *Daxx* also safeguard pancreatic tissue from inflammation [18,36]—as their loss resulted in an inability to restore tissue homeostasis following caerulein-induced pancreatitis—and endocrine fitness [34]—as *Atrx* loss caused increased weight gain and endocrine dysfunction. However, the mechanisms leading to the metabolic dysfunction are unknown and these very interesting and novel findings prompted us to validate our results with a distinctive approach.

During the last four years, we have been working on a GEMM of β cell-specific knockout (KO) of *Atrx* using the *Pdx1-Cre* system (*P.Atrx*). *Rip-Cre* lines, although broadly used, have been solidly linked to endocrine dysfunction [37]. Recently, aristaless-related homeobox (ARX) and pancreatic and duodenal homeobox 1 (PDX1), drivers of α and β cell differentiation, respectively, have been emerging as novel genetic signatures of human PanNET classification. We now know that the most altered endocrine cell population in *ATRX*/*DAXX*-mutated PanNETs harbours a typical landscape signature of α cell (*ARX*^+^/*PDX1*^-^) [38,39]. We and others have confirmed that *Atrx* is not a robust tumour suppressor in mouse endocrine pancreas by using *Rip-Cre* [34] and *Pdx1*-*Cre* [35]. Following our report on the involvement of Atrx in the homeostasis of the endocrine function using the *Rip-Cre* system, we decided to study for the first time, in a *Pdx1*-driven context, the impact of the *Atrx* disruption in the putative endocrine function impairment, as well as readdress its role in PanNET formation. By using a Cre-driver that is harmless to endocrine fitness, we are also circumventing the inherent phenotype of *Rip-Cre* in this context [37]. We verified that by using a different promoter, the impact of *Atrx* loss in endocrine dysfunction and weight gain became more pronounced. In this study, we present evidence of *Atrx* playing a role in endocrine homeostasis, with mice showing a diabetic state and pancreatic fat infiltration.

## 2. Materials and Methods

### 2.1. GEMM Generation

The *Atrx^y/f^* male and *Atrx^f/f^* female (*floxed*) mice were a kind donation from Douglas R. Higgs and backcrossed with C57BL/6 (B6). These mice carry a flanked *Atrx* gene with a *floxed* neo^r^ cassette inserted within intron 17, and *loxP* sites flanking exon 18 [40,41,42]. *B6.FVB-Tg(Pdx1-Cre)6Tuv/J* mice were obtained from the Jackson Laboratory [43] and crossed with the *Atrx floxed* mice to generate *Pdx1-Cre^+/−^;Atrx^y/wt^* and *Pdx1-Cre^+/−^;Atrx^wt/wt^* male and female controls (*P.Atrx^WT^*), *Pdx1-Cre^+/−^;Atrx^y/f^* and *Pdx1-Cre^+/−^;Atrx^f/f^* male and female homozygous individuals (*P.Atrx^HOM^*), and *Pdx1-Cre^+/−^;Atrx^f/wt^* heterozygous females (*P.Atrx^HET^*). In the absence of statistical differences, the *P*.*Atrx^HOM^* and *P*.*Atrx^HET^* genotypes were sometimes analysed together in the same genotype group (“*P.Atrx^KO^*”). Graphical representations of the *P.Atrx^WT^*, *P.Atrx^HET^*, *P.Atrx^HOM^*, and *P.Atrx^KO^* genotype groups are given by a different colour scheme (green, teal, ocean blue, and light blue). Animal genotyping was performed as previously reported (Supplementary File S1: Table S1) [34]. Mice were rederived once during the project to ensure the absence of disease-causing pathogens.

The in vivo studies were performed following the Portuguese National Regulation established by Decreto-Lei n.° 113/2013, the national transposition of the European Directive 2010/63/EU for the Care and Use of Laboratory Animals. Procedures were submitted for evaluation and approved by the i3S Animal Welfare and Ethics Review Body and the Portuguese National Authority for Animal Health (DGAV)-project license code 13020/2017-05-08. Procedures were carried out by FELASA C-certified operators (TBG and SM). Mice were bred and maintained at the animal facility of i3S, accredited by the Association for Assessment and Accreditation of Laboratory Animal Care (AAALAC), under a standard 12 h light/dark cycle, with water and ad libitum chow diet (Tecklad Global Diet Rodent 2014S, Envigo, Indianapolis, IN, USA).

### 2.2. Animal Husbandry and Longitudinal Follow-Up

A cohort with ageing mice was followed for up to 24 months (mo.). During the project, animals were subjected to different and complementary in vivo manipulations; procedures, animal sampling, and the sex and genotype data acquired are presented in Supplementary File S1: Table S2, following the ARRIVE 2.0 checklist [44].

An equal strategy, as previously reported [34], at predetermined time points included data acquisitions for the following parameters: monthly longitudinal weighing, glycaemia evaluation, intraperitoneal glucose tolerance tests (ipGTTs), and hemograms. A longitudinal fashion of data collection was, when possible, favoured; for data analysis, both longitudinal and unpaired data were used at all times. Regarding the histopathological (HP) evaluation, euthanasia dates were distributed to represent of all age groups by genotypes, being composed of eight age groups (3, 6, 9, 12, 15, 18, 21, and 24 mo.) For data analyses, age formula and age range (calculated from dates of birth (DOB) and dates of procedure (DOP) or death (DOD)) were considered, as previously described [34]. No data were collected from animals that reached a humane endpoint (HEP). The HEP was defined as a gradual weight loss over three consecutive weighings, evidence of dehydration and lethargy, and reluctance to move when stimulated. The clinical evaluation of mice was performed by animal facility staff and the researcher T.B.G. In all the above-stated analyses, a single animal was our experimental unit. Sample size estimations were calculated in G*Power 3.1.9.2.

### 2.3. Euthanasia and Organ Collection

Euthanasia was conducted for organ collection and HP evaluations of all age groups. Generally, animals were euthanised via an IP injection of ketamine (150 mg/kg) and medetomidine (2 mg/kg), followed by death confirmation with cervical displacement. Exsanguination allowed blood collection for hemogram analysis and ELISA assay. The pancreas and liver were collected from all necropsied animals; pancreata were collected in the block with duodenum, stomach, spleen, and abdominal fat to preserve their physiological diffuse mesenteric distribution [45]. For each animals’ necropsy, a complete macroscopic form was created and disclosed other organs that exhibited pathological alteration.

### 2.4. Histopathological Evaluation and Immunohistochemistry Assays

Tissue fixation was prepared in 4% paraformaldehyde (pH 7.4) for 24 h and routinely processed in an automatic tissue processor. Fixed and paraffin-embedded tissue sections with a 4 µm thickness were obtained and stained with haematoxylin and eosin (H&E).

HP evaluation was conducted by human and veterinarian pathologists (T.B.G., I.B., and S.C) using a previously determined score [34]. The scoring system of inflammatory lesions in the pancreas included the following parameters: (1) oedema, (2) fibrosis, (3) loss of lobular pattern, (4) duct/vessel dilation, (5) focal acinar atrophy, (6) peripancreatic chronic inflammation (CI), (7) acinar CI, (8) periductal/perivascular (Pd/Pv) CI, (9) intra-/peri-islet CI, (10) intrapancreatic fatty infiltration (FI), and (11) peripancreatic fat deposition. Except for FI, the parameters mentioned above were evaluated in a four-level scoring system, from 0 (<5% altered) to 1 (low-grade lesion, 5–33% altered), 2 (moderate-grade lesion, 33–66% altered), or 3 (high-grade lesion, >66% altered); FI was classified as 0 (<5% altered), 1 (low-grade lesion, 5–10% altered), 2 (moderate-grade lesion, 10–20% altered), or 3 (high-grade lesion, >20% altered). The sum of the first nine parameters obtained the HP score, and the sums of the most prevalent parameters were evaluated separately, namely Pd/Pv CI, as previously described [34]. The parameters FI and FR were evaluated individually and summed. The presence of ductal dysplasia was also qualitatively determined; the sum of duct/vessel dilation, focal acinar atrophy, and ductal dysplasia obtained the ageing score.

Liver tissue was evaluated using the non-alcoholic fatty liver disease (NAFLD) activity score (NAS) as previously reported [46,47]. The following parameters were considered: (1) microvesicular steatosis, (2) macrovesicular steatosis, (3) hypertrophy, (4) lobular inflammatory foci (all lymphocytic foci except at portal location), (5) portal inflammation, (6) microgranulomas, and (7) oedema. All parameters were also evaluated as previously described [34], and macrovesicular steatosis was evaluated separately.

The differential diagnosis of tumour sections was evaluated using a panel of pan-cytokeratins, vimentin, and CD45 antibodies to discriminate the most probable phenotype of undifferentiated lesions. A list of antibodies and specifications of the respective IHC assays is presented in Supplementary File S1: Table S3.

### 2.5. Blood Collection and Hemogram Analyses

For the genotypes and age groups (3, 6, and 12 mo.), the hemograms analyses were performed from longitudinal blood collections and as previously described [34].

### 2.6. Glycaemia Assessment and Glucose Tolerance Tests

Glycaemia assessments were assessed by a unique operator (T.B.G.) according to the current guidelines [48]. Unpaired and longitudinal single glycaemia measurements and ipGTTs were performed in all genotype groups at 3, 6, and 12 mo., as previously described [34]. Glycaemia cut-off values were considered prediabetic when above 150 mg/dL and diabetic when above 240 mg/dL.

### 2.7. Endocrine Fraction Evaluation

H&E-stained slides scanned at 40× magnification (226 nm/pixel resolution) in NanoZoomer S60 (Hamamatsu, Hamamatsu, Japan) were evaluated by a deep learning algorithm trained in HALO^®^ Image Analysis Platform version 3.5.3577 (Indica Labs Inc., Albuquerque, NM, USA). The algorithm was trained to segment pancreatic islets, the exocrine portion, and the lymphocytic infiltration. Representative images of the markups containing the ‘endocrine’, ‘exocrine’, and ‘lymphocyte’ classes are available in Supplementary File S1: Figure S1. The segmentation output was post-processed in Fiji [49], and a set of measurements was extracted for the morphological characterisation of pancreatic islets, as previously reported [34]. Male and female mice were analysed together, and the results were organised by four age groups (3, 6, 12, and 18–24 mo.).

### 2.8. ELISA Immunoassay

Serum insulin quantification was performed using the Mouse Insulin ELISA Kit (RAB0817, Millipore, Burlington, MA, USA), following the manufacturer’s recommendations.

### 2.9. Statistical Analysis

Statistical analyses were conducted on Prism 9 for macOS (Version 9.1.1) or IBM SPSS Statistics (Version 28.0). Significancy between groups was considered at a *p*-value < 0.05. The outliers’ ROUT (1%) method was applied in the weight and glycaemia analyses. In all graphs *, **, ***, and **** correspond to *p*-values < 0.05, <0.01, <0.001, and <0.0001, respectively. For inter-parameter comparisons, Pearson’s was used when *n* ≥ 15 or Kendall’s correlations if *n* < 15 in at least one comparison group (Supplementary File S2: Table S3); interpretation of the correlation coefficients was presented as Schober et al. [50] (0.10–0.39 negligible, 0.40–0.69 moderate, 0.70–0.89 strong, and 0.90–1.00 very strong correlation).

## 3. Results

### 3.1. Study Population

**Figure 1:**
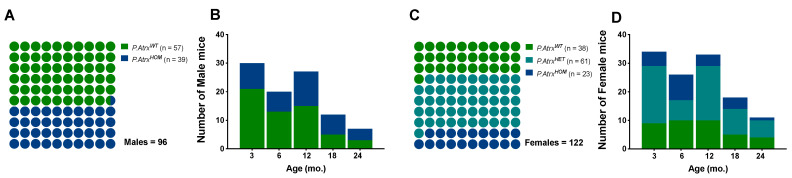
Study population. The proportion of genotypes between male (**A**,**B**) and female mice (**C**,**D**), with the respective distribution by the five age groups. Green means *P.Atrx^WT^*, teal means *P.Atrx^HET^*, and ocean blue means *P.Atrx^HOM^* (**A**–**D**). Mice’ ages were calculated by the time of death.

In this study, a population of 218 mice (96 males and 122 females) was euthanised at different time points between 2018 and 2022. At the time of euthanasia (date of death, DOD) the mice’ age was similar between sex groups (mean ± standard deviation (SD)): 9.0 ± 6.2 mo. in males and 9.6 ± 6.3 mo. in females; age variation was also balanced among genotypes: minimum age from 1.7 to 1.9 mo., and maximum ages from 24.4 to 24.3 mo. in males and females, respectively. The *P.Atrx^HOM^* genotype was represented in a proportion of 1.0:1.5 and 1.0:1.7 to *P.Atrx^WT^* controls in males and females, respectively; the *P.Atrx^HET^* genotype was restricted to females and at a higher proportion to allow more robust comparisons with *P.Atrx^HOM^* individuals (Figure 1).

### 3.2. Atrx Disruption at β Cells Enhanced Pancreatic Fat Accumulation and Did Not Cause Pancreatic Neuroendocrine Tumours

**Figure 2:**
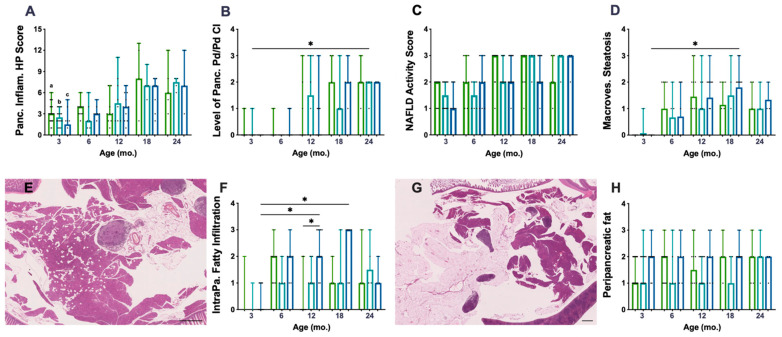
Inflammatory and fat accumulation in the pancreas and hepatic steatosis. There are no differences among genotypes concerning pancreatic inflammation score (**A**) nor in periductal/perivascular (Pd/Pv) chronic inflammation (CI) specifically (**B**). However, *P.Atrx^HET^* females present a higher inflammation score, mostly due to Pd/Pv CI, by 12 mo. (**A**,**B**). There are no differences among the genotypes in alcoholic fatty liver disease (NAFLD) activity score (NAS) (**C**) nor in macrovesicular steatosis specifically (**D**); however, *P.Atrx^HOM^* presents higher macrovesicular steatosis compared with age-matched controls by 12 and 18 mo. Intrapancreatic fatty infiltration (FI), defined by scattered vacuoles within the exocrine pancreas (**E**), is significantly higher in 12- and 18-mo.-old *P.Atrx^HOM^* than age-matched controls (**F**). The extent of peripancreatic fatty tissue around pancreatic lobes (**G**) is constant (and high) in *P.Atrx^HOM^* of all ages, only increasing in comparison to age-matched controls by 3 and 12 mo. Ageing significantly enhances FI in *P.Atrx^HOM^* but does not allow discrimination of genotypes concerning peripancreatic fat deposition (**H**). Results are shown as the median ± range values (**A**–**D**,**F**,**H**). * *p* < 0.05, ** *p* < 0.01. a * *P.Atrx^WT^* (3 mo. vs. 18 mo. and 24 mo.) (**A**), b * *P.Atrx^HET^* (3 mo. vs. 18 mo. and 24 mo.) (**A**), c ** *P.Atrx^HOM^* (3 mo. vs. 18 mo. and 24 mo) (**A**). Green means *P.Atrx^WT^*, teal means *P.Atrx^HET^*, and ocean blue means *P.Atrx^HOM^* (**A**–**D**,**F**,**H**).

The histopathological (HP) scoring system used to characterise chronic inflammation (CI) lesions was performed on 136 mice pancreata. *P.Atrx^KO^* mice did not present an increased HP score compared to age-matched controls (Figure 2A). The Pd/Pv CI, previously described as responsible for the HP score increase, was similar among genotypes (Figure 2B), despite the augmented Pd/Pv CI level in *P.Atrx^HET^* females by the age of 12 mo. All genotypes’ pancreatic inflammation increased with ageing (Figure 2A,B), but statistical significance was not reached. When stratifying the different lesions by the three age groups encompassing all animals (3 mo., 6–12 mo., and 15–24 mo.), it was noticeable that, from 6 mo., *P.Atrx^KO^* presented a slightly increased percentage of moderate- and marked-grade pancreatic CI lesions in comparison to age-matched controls (Supplementary File S2: Figure S2). Additionally, 15–24-mo.-old *P.Atrx^KO^* mice presented an increased ageing score than age-matched *P.Atrx^WT^* (Supplementary File S2: Figure S2).

To determine if increased weight profiles could contribute to increased pancreatic CI lesions, we matched weights and pancreatic HP and found a positive and moderate correlation (r = 0.651) between *P.Atrx^HOM^* weights and the pancreatic HP score at 18 mo. (Supplementary File S2: Table S3).

When evaluating the non-alcoholic fatty liver disease (NAFLD) activity score (NAS) as an essential feature of ageing, *P.Atrx^KO^* mice did not show any significant increase in NAS when compared to age-matched controls (Figure 2C). However, when specifically analysing the macrovesicular steatosis, it was observed that *P.Atrx^HOM^* mice between 12 and 18 mo. exhibited an increased score than age-matched controls (Figure 2D). Moreover, unlike with NAS, the ageing-increased macrovesicular steatosis was more easily perceived and statistically significant between 3- and 18-mo.-old *P.Atrx^HOM^* individuals. Positive and moderate correlations were found between NAS and the weights of 12-mo.-old *P.Atrx^WT^* (τ = 0.584) and 12- and 18-mo.-old *P.Atrx^HOM^* (τ = 0.520 and τ = 0.569, respectively). More detailed information about the evolution of NAS over time and its correlations with weight gains can be found in Supplementary File S2: Table S3.

The histopathological evaluation of pancreata showed a preponderant presence of fat vacuoles, either scattered throughout the exocrine pancreas (Figure 2E)—defining *intrapancreatic fatty infiltration* (FI)—or surrounding pancreatic tissues mainly in the form of mesenteric fat (Figure 2G). Concerning FI, it was observed that *P.Atrx^HOM^* mice presented an increased score by 12 and 18 mo., statistically significant by 12 mo. (Figure 2F); ageing significantly contributed to the increase in this parameter. Concerning FR, only by the age of 3 and 12 mo., *P.Atrx^HOM^* mice presented an increased score compared to age-matched controls; of note, the FR levels were constant in *P.Atrx^HOM^* throughout all age groups, as ageing did not seem to influence this parameter (Figure 2H); by 24 mo., all genotypes presented the same score. Summarising FI and FR, it was observed that *P.Atrx^KO^* mice belonging to the 6–12-mo. and 15–24-mo. age groups had higher levels than age-matched controls (Supplementary File S2: Figure S2). Representative images of the pancreas not infiltrated with fat (FI score 0 and FR score 0) are shown in Supplementary File S2: Figure S3.

By the time of necropsy, macroscopic evaluation denoted a slight increase in the prevalence of hepatomegaly and splenomegaly (17% and 11% increase, respectively) when comparing *P.Atrx^KO^* mice aged 6–12 mo. with age-matched *P.Atrx^WT^* mice (Supplementary File S2: Figure S3).

In all genotypes, 29 tumours were detected, with 22 being malignant, composed of primary or secondary tumours, and then evaluated to determine the most likely phenotype. Lymphomas were the most common phenotype found in *P.Atrx^WT^* mice (75%), whereas, in the *P.Atrx^KO^* genotype, most tumours were mesenchymal (40%), followed by lymphomas (30%). Tumour incidence was higher in *P.Atrx^KO^* female mice (*P.Atrx^WT^* vs. *P.Atrx^KO^*; 50% vs. 50%, respectively) but decreased in male mice (*P.Atrx^WT^* vs. *P.Atrx^HOM^*; 64% vs. 36%, respectively) (Supplementary File S2: Figure S4). Pancreatic tumours (*n* = 4) were found in *P.Atrx^KO^* and *P.Atrx^WT^* mice with equally distributed phenotypes (50% mesenchymal, 50% lymphoma); representative images are presented in Supplementary File S2: Figure S5. Liver tumours (benign and malignant) presented equal occurrence and were equal between *P.Atrx^WT^* and *P.Atrx^KO^*.

Like in our previous model [34], we questioned if the exacerbated pancreatic inflammatory lesions would be detected systemically in a longitudinal hemogram evaluation: (a) the total white blood cell (WBC) count, *P.Atrx^KO^* mice did not disclose different levels compared to age-matched controls; (b) the lymphocyte percentage, which was not different in *P.Atrx^KO^* individuals compared to age-matched controls; and (c) the neutrophil-to-lymphocyte ratio (NLR), assessed due to its potential prognostic relevance in anticipating malignancy, did not differ between genotypes (Supplementary File S2: Figure S6). The complete haematological reports of *P.Atrx* mice are in Supplementary File S2: Table S4.

### 3.3. P.Atrx^KO^ Mice Exhibited Increased Weight Gains and Glycaemia Levels since 3 mo.

**Figure 3:**
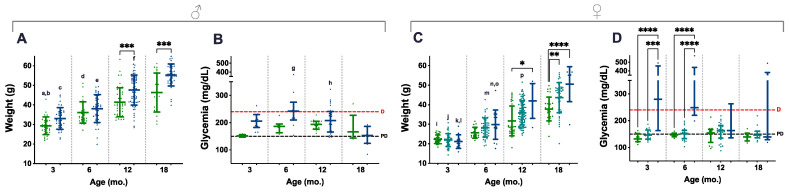
Weight and glycaemia. Weight and glycaemia evaluations demonstrate that *P.Atrx^KO^* male (**A**,**B**) and female (**C**,**D**) mice present increased weight gains and glycaemia levels than age- and sex-matched controls. *P.Atrx^HOM^* males and females are significantly heavier than age- and sex-matched controls by 12 mo. and 18 mo. (**A**,**C**, respectively). *P.Atrx^HET^* females also present a significantly higher weight than age-matched controls by 18 mo. (**C**). *P.Atrx^HOM^* males and females show increased glycaemia than age- and sex-matched controls up to 12 mo.; while males present median levels above diabetic level only by the age of 6 mo. (*p* = 0.07) (**B**), females show such a trend by 3 and 6 mo. old, with statistical significance (**D**). Results are shown as the mean ± SD (**A**,**C**) and median ± interquartile range (**B**,**D**). Age groups of 9 and 15 mo. were included in the 12 and 18 mo. age groups, respectively (**A**–**D**). Inter-genotype differences are shown by: * *p* < 0.05, ** *p* < 0.01, *** *p* < 0.001, and **** *p* < 0.0001. Ageing-related comparisons are given by small letters: a ** *P.Atrx^WT^* (3 mo. vs. 6 mo.), b **** *P.Atrx^WT^* (3 mo. vs. 12 mo. and 18 mo.), c **** *P.Atrx^HOM^* (3 mo. vs. 12 mo. and 18 mo.), d **** *P.Atrx^WT^* (6 mo. vs. 18 mo.), e **** *P.Atrx^HOM^* (6 mo. vs. 12 mo. and 18 mo.), f **** *P.Atrx^HOM^* (12 mo. vs. 18 mo.); g **** *P.Atrx^HOM^* (6 mo. vs. 18 mo.), h * *P.Atrx^HOM^* (12 mo. vs. 18 mo.), i **** *P.Atrx^WT^* (3 mo. vs. 12 mo. and 18 mo.), j **** *P.Atrx^HET^* (3 mo. vs. 6 mo., 12 mo., and 18 mo.), k * *P.Atrx^HOM^* (3 mo. vs. 6 mo.), l **** *P.Atrx^HOM^* (3 mo. vs. 12 mo. and 18 mo.), m **** *P.Atrx^HET^* (6 mo. vs. 12 mo. and 18 mo.), n ** *P.Atrx^HOM^* (6 mo. vs. 12 mo.), o **** *P.Atrx^HOM^* (6 mo. vs. 18 mo.), and p **** *P.Atrx^HET^* (12 mo. vs. 18 mo.). Green means *P.Atrx^WT^*, ocean blue means *P.Atrx^HOM^* (**A**–**D**), and teal means *P.Atrx^HET^* (**C**,**D**). D diabetic, PD prediabetic.

A total of 772 weighings, from 217 animals, were considered and distributed along four age groups (3, 6, 12, and 18 mo.); all the available non-longitudinal weights obtained during DOD were also included. Blood glucose levels were obtained at the same time points, and 207 measurements (from 107 animals) were used for data analysis. Ageing contributed to increasing weights in mice of all genotypes and the difference was statistically significant, especially during the first 12 mo. (Figure 3A,C, letters). In contrast, genotype-related differences were already perceived in 3- and 6-mo. male mice, but statistical significance was only present in the 12- and 18-mo. age groups of both males and females, as *P.Atrx^HOM^* individuals consistently presented higher weights than age-matched controls (Figure 3A,C, asterisks); during this period, most *P.Atrx^HOM^* presented a median weight corresponding to overweight or obesity.

In parallel, *P.Atrx^KO^* mice of both sexes also tended to present increased glycaemic levels. However, such endocrine dysfunction was only marked in the 3- and 6-mo. age groups, higher in *P.Atrx^HOM^* than age-matched controls (Figure 3B,D). Nevertheless, statistical significance was only obtained in females, as the *P.Atrx^HOM^* genotype developed significantly higher glycaemia levels, at 3 mo. and 6 mo. of age, within levels compatible with diabetes (Figure 3D). In the correlation analysis, weights positively correlated with glycaemia in the *P.Atrx^WT^* genotype by 6 and 12 mo. (r = 0.718 and r = 0.397, respectively) and with NAS in *P.Atrx^WT^* and *P.Atrx^HOM^* mice (τ = 0.584 and τ = 0.520) by the age of 12 mo. Moreover, by 18 mo., the weights positively correlated with pancreatic HP and hepatic NAS scores in *P.Atrx^HOM^* groups (τ = 0.651 and τ = 0.569) (Supplementary File S2: Table S3).

### 3.4. P.Atrx^KO^ Mice Show Improper Ageing-Related Growth of the Endocrine Fraction and Similar Fasted Insulinaemia

**Figure 4:**
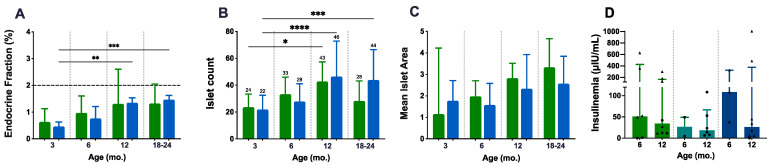
Endocrine fitness: endocrine fraction, islet count, mean islet area, and insulinaemia. Both genotype groups present an age-related increase in endocrine fraction (EF), islet count, and mean islet area (**A**–**C**). The mean islet area shows a preponderance of *P.Atrx^WT^* mice over *P.Atrx^KO^* (**C**). Insulinaemia assessments do not retrieve any genotype-related difference; in all genotype groups, the insulinaemia decreases from 6 mo. to 12 mo. Of note, *P.Atrx^HOM^* presents higher basal levels by 6 mo. (**D**). Age groups of 9, 15, and 21 mo. were included in 12 and 18–24 mo., respectively (**A**–**D**). Results are shown as the median ± IQR (**A**,**C**,**D**) and mean ± SD (**B**) * *p* < 0.05, ** *p* < 0.01, *** *p* < 0.001, and **** *p* < 0.0001. Green means *P.Atrx^WT^* (**A**–**D**), which was compared with the *P.Atrx^KO^* genotype (**A**–**C**), represented with a light blue, and with the *P.Atrx^HET^* and *P.Atrx^HOM^* genotypes coloured with teal and ocean blue, respectively (**D**). Triangles and circles are used to identify males and females, respectively.

A detailed morphological characterisation of pancreatic islets was performed to complement the weight and glycaemia analysis and as a readout of in situ endocrine fitness (Figure 4A–C). The endocrine fraction (EF) was quantified in HE-stained slides following segmentation of the endocrine and exocrine portions using HALO^®^ software. As age progressed, the EF increased slightly and at a constant rate in both *P.Atrx^WT^* and *P.Atrx^KO^* mice, being statistically significant in *P.Atrx^KO^* individuals (Figure 4A); however, no differences in the EF between *P.Atrx^KO^* and *P.Atrx^WT^* mice were detected. Comparing the ageing-related increase of EF between 3 and 24 mo., compared to 24-mo.-old *P.Atrx^WT^* (1.71%, median value), age-matched *P.Atrx^KO^* presented a similar but diminished fraction (1.49, 6% less). Islet numbers in both *P.Atrx^WT^* and *P.Atrx^KO^* mice equally increased with ageing, being statistically significant in both genotypes (Figure 4B); however, islet numbers at 18–24 mo. tended to be lower than 12-mo.-old counterparts of both genotype groups. The mean islet area (given by EF/islet count) was tendentially higher in *P.Atrx^WT^* than in *P.Atrx^KO^* mice (Figure 4C).

Aiming to further characterise the endocrine metabolism disturbance in *P.Atrx^KO^* mice, we used ELISA assays to quantify the insulinaemia in non-fasted and fasted mice of all genotypes (Figure 4D). Although a tendency was noted in all genotype groups, with insulinaemia decreasing from 6 mo. to 12 mo., *P.Atrx^HOM^* mice presented higher basal levels by 6 mo. than the age-matched controls and *P.Atrx^HET^*; the assay results in Figure 4D analyses both genders, while in Supplementary File S2: Figure S7, the evaluation of non-fasted and fasted insulinaemia values analysed separately are available for consultation. By pooling the results of each genotype regardless of mice age, the level of insulin was higher in the *Atrx^HOM^* group (mean ± S.E., 188.80 ± 94.40 µIU/mL, *n* = 11 male and female mice), followed by *Atrx^WT^* male and female controls (128.90 ± 49.05 µIU/mL, *n* = 14), and by *Atrx^HET^* females (33.45 ± 12.62 µIU/mL, *n* = 8). The insulin resistance (IR), inferred by glycaemia/insulinaemia ratios, were similar, although slightly higher, in 12 mo. *Atrx^HOM^* male and *Atrx^HET^* female mice than age-matched controls (Supplementary File S2: Figure S7).

### 3.5. P.Atrx^HOM^ Mice Exhibit Frank Glucose Intolerance by 3 mo.

**Figure 5:**
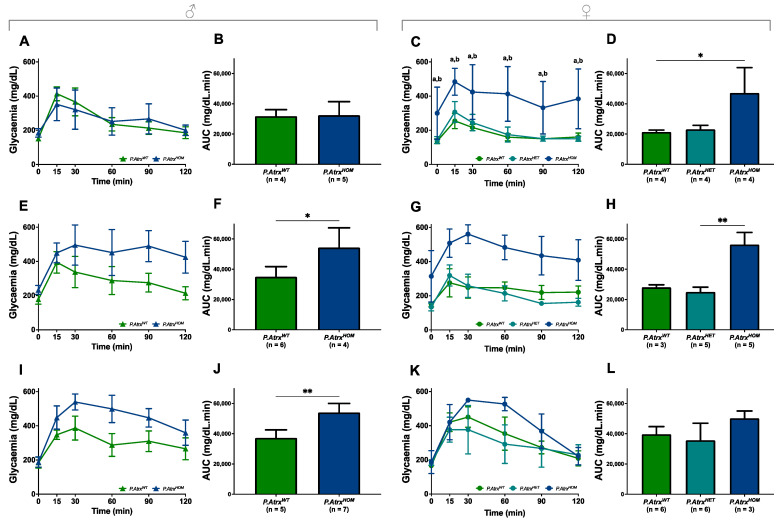
Evidence of glucose intolerance. Intraperitoneal glucose tolerance tests (ipGTTs) performed in 3-mo.- (**A**–**D**), 6-mo.- (**E**–**H**), and 12-mo.-old mice (**I**–**L**). By 3 mo., *P.Atrx^HOM^* females have significantly increased glucose intolerance, whereas males do not (**A**–**D**). By 6 mo., *P.Atrx^HOM^* males already have significantly increased glucose intolerance (**E**,**F**), while females show a similar tendency (**G**,**H**); by 12 mo., *Atrx^HOM^* males preserve the significant deterioration of glucose tolerance (**I**,**J**), whereas females of all genotypes exhibit equivalent GTT-AUC values (**K**,**L**). * *p* < 0.05, ** *p* < 0.01, **** *p* < 0.0001. Two-way ANOVA was used for comparisons in each time point: **a** **** *p* < 0.0001 (*P.Atrx^WT^* vs. *P.Atrx^HOM^*) (**C**), **b** **** *p* < 0.0001 (*P.Atrx^HET^* vs. *P.Atrx^HOM^*) (**C**).

ipGTTs were performed at 3, 6, and 12 mo. for both genders and for all genotypes to evaluate the role of Atrx loss in endocrine dysfunction. By 3 mo., male mice of all genotypes presented a comparable response to glucose administration, as they could gradually restore normoglycaemia within 60 min (Figure 5A,B). In contrast, by the same age, *P.Atrx^HOM^* female mice already exhibited significantly increased glucose intolerance (Figure 5C,D). By 6 mo., both *P.Atrx^HOM^* male and female mice exhibited a significantly higher glucose intolerance than age-matched controls (Figure 5E–H); by 12 mo., such imbalance is maintained in *P.Atrx^HOM^* of both sexes (Figure 5I–L), although the significance was lost in females due to the increased GTT-AUC of *P.Atrx^W^*^T^; the evolution of GTT-AUC over time in all genotypes is available in Supplementary File S2: Figure S8.

Moreover, in the correlation analysis, GTT-AUC positively correlated with weight by 12 mo., but solely in the *P.Atrx^WT^* genotype (τ = 0.477) (Supplementary File S2: Table S3).

## 4. Discussion

PanNETs are rare and clinically challenging entities. During the last 15 years, their genetic profile has been progressively unveiled as high-throughput sequencing techniques have emerged, and *ATRX* and *DAXX* mutations were reported in PanNETs (11–17% and 14–25%, respectively) [22,23,24]. In addition to *MEN1* inherited and acquired mutations, the loss of *ATRX* or *DAXX* was indicated to be the primary driver of somatic events in PanNET biology through epigenetic changes [51,52,53]. Evidence from extensive PanNET cohorts has ascertained the prognostic value of *ATRX*/*DAXX* mutations [27,28,29,31,32]. In particular, the apparent correlation between chromatin remodellers’ loss of function and ALT activation has emerged as a reliable indicator of poor prognosis, helpful in identifying “high-risk” PanNET patients [54].

We still have limited knowledge concerning the contribution of the newly identified genes to tumour initiation and progression. Knowing the mechanisms involved in *ATRX*/*DAXX* inactivation and the initiation of ALT will likely clarify the pertinence of their clinical implications [55]. We believe that suitable GEMMs addressing these issues will help down the road.

Recently, we developed and studied a model generated using the *Rip-Cre* system to conditionally target *Atrx* disruption to pancreatic β cells (*R.Atrx*) and reported for the first time that the conditional disruption of *Atrx* in mice pancreas is not a sufficient event to drive PanNET formation [34]; other authors have reported similar findings afterwards [35]. Instead, the *Atrx* disruption at endocrine islets seems to play a role in anticipating and aggravating inflammageing and ageing-related deterioration of endocrine functions [34]. *R.Atrx^HOM^* mice exhibited higher glycaemia levels (prediabetic) and glucose intolerance with sexual dimorphism (detected earlier in males) [34]. In 2019, it had already been reported that the loss of *Atrx* enhanced pancreatic injury and susceptibility to KRAS-mediated (*M.K.Atrx*) pancreatic damage in female mice, pioneering the attribution of anti-inflammatory and tumour-suppressive roles to *Atrx* [36] (Table 1).

Considering the relevance of *ATRX* in sporadic human PanNETs and the scarcity of GEMMs addressing its role in endocrine function, we studied a mouse model that conditionally targets *Atrx* at pancreatic β cells expressing *Cre* under the pancreatic and duodenal homeobox 1 (*Pdx1*) promoter (*P.Atrx*). By applying the same methodology as before [34], we primarily aimed to assess the putative role of *Atrx* heterozygous and homozygous loss (*P.Atrx^KO^*) as a disruptor of endocrine fitness and putative PanNET driver event.

We started analysing the histopathological (HP) alterations of the pancreas and liver. We observed that no apparent genotype-related differences in the overall pancreatic HP score were perceived. However, by 12 mo., *P.Atrx^HET^* females presented a higher HP score and a higher periductal/perivascular (Pd/Pv) score. These results are, in part, comparable to those obtained from *R.Atrx^HET^*, which presented the highest Pd/Pv levels by 9 mo., contributing to the rising of the pancreatic HP score [34]. Like before, as ageing progressed, the differences among genotypes seemed to be harder to detect. When looking at NAS, as the liver is an organ sensitive to inflammageing, we observed that losing *Atrx* did not aggravate the overall hepatic steatosis levels. Still, when only analysing NAS parameters, we detected that macrovesicular steatosis alone could discriminate mice by genotype; we observed that, although not significant, *P.Atrx^HOM^* mice presented increased levels of macrovesicular steatosis than age-matched controls by 12 and 18 mo. Most mice presented pancreatic scattered fat accumulation, an alteration we termed *intrapancreatic fatty infiltration* (FI), also designated by *fatty pancreas* [56]; we decided to quantify this alteration and observed that FI significantly increased with ageing and that *P.Atrx^HOM^* mice significantly presented more adipocyte deposition within pancreatic tissue than age-matched controls by 12 and 18 mo. In many slides, it was noted that the pancreatic lobes were surrounded by peripancreatic fatty tissue, which was also quantified. It remains to be disclosed if, to some extent, this fatty tissue is replacing pancreatic lobes, a situation termed *pancreatic fatty replacement* (FR). Contrary to FI, the peripancreatic fat extension did not alter much with ageing; *P.Atrx^HOM^* mice exhibited the same median levels over time, higher than age-matched controls by 3 and 12 mo.

Weight profiles help explain all these results. As expected, mice weights constantly increased over time in all genotypes; however, genotype-related differences were noticed with a mild sexual dimorphism. Both males and females with *Atrx* homozygous deletion presented increased weights than age-matched controls, such difference was statistically significant by 12 and 18 mo. Nevertheless, while the mean weight of the male mice was already higher than controls by 3 mo., more time is required to confirm such genotype-related differences in females. *P.Atrx^HET^* seem to have mean weights between *P.Atrx^WT^* and *P.Atrx^HOM^*. Male and female mice (controls and KO) presented weights compatible with overweight or obese measurements by 3 mo. or 6 mo., respectively, and kept progressing with ageing, especially in males; such augmented weights and the subsequent fat accumulation likely hampered discriminating the genotype-related differences in pancreatic HP and NAS scores.

Evaluating longitudinally glycaemias revealed that *P.Atrx^HOM^* males and females presented increased glycaemias than age-matched controls starting at 3 mo.; such hyperglycaemic values were within diabetic values (i.e., above 240 mg/dL) in females (3 mo.) sooner than in males (6 mo.). ipGTT tests were subsequently performed and reinsured the sexual dimorphism of endocrine dysfunction onset: *P.Atrx^HOM^* females presented obvious glucose intolerance by 3 mo., which was maintained until 6 mo., but less pronounced by 12 mo.; in contrast, *P.Atrx^HOM^* males were tolerant to glucose administration by 3 mo., but intolerant in the 6- and 12-mo. age groups.

As a readout of endocrine fitness, an endocrine fraction measurement and insulinaemia quantification using ELISA assay were performed. Neither evaluation highlighted marked differences between *P.Atrx^WT^* and *P.Atrx^KO^*. The main motive behind the choice of another promoter rather than the rat insulin promoter (*Rip*) was the reports of the prudent use of *Rip-Cre* driver lines, whose main side effects are a leaky expression in the hypothalamus [57,58] and the spontaneous early development of glucose intolerance and impaired insulin secretion (before two months) [59,60]. By using the *Pdx1-Cre* system, we could study the effects of losing *Atrx* in the endocrine pancreas regardless of the putative influence of the *Rip-Cre* system. Unexpectedly, besides the early-onset of overweight and obese measurements, *P.Atrx^WT^* and *P.Atrx^KO^* mice did not double their EF as their age progressed as expected [61]; their EF remained constant from 12 mo., consistently below 2%, the described value for most mammalian species [62]. Since the quantification method is the same as the one used in our previous study [34], we believe that, contrarily to the non-obese *R.Atrx* mice, the increased peripancreatic fat deposition and (consequent) putative FR in the *P.Atrx* model may be deflating this parameter in the context of overweight and obese weight measurements. Moreover, we cannot exclude a putative contribution of Atrx loss in exocrine tissue that could, in turn, predispose an acinar counterpart to FI/FR.

Concerning the insulinaemia results, we find them comparable to the *R.Atrx* mice, as the *P.Atrx^WT^* and *P.Atrx^KO^* age groups presented decreased insulinaemia values by 12 mo. compared to genotype-related 6-mo.-old individuals; peripheral insulin resistance (IR), given by the glycaemia to insulinaemia ratio, was also higher at 12 mo. than 6 mo. in both genotype groups. *P.Atrx^HOM^* mice presented the highest insulinaemias regardless of mice age and developed the highest IR by 12 mo. Of note, the IR values were over two-fold the *R.Atrx^KO^*, probably due to their substantially higher weights.

We developed an obese diabetic GEMM. Obesity is a preponderant factor in inflammageing onset and progression. Lipid accumulation, accentuated by ageing, leads to obesity, type 2 diabetes (T2D), and dyslipidaemia; these have been set as direct contributors to the *fatty pancreas*, i.e., intrapancreatic fatty infiltration [56]. FI causes a release of cytokines, adipokines, and pro-inflammatory factors responsible for the progression of pancreatic intraepithelial neoplasia (PanIN) lesions to PDAC [63].

Daxx has been implicated in endogenous retroviral element (ERV) silencing by safeguarding chromatin through epigenetic mechanisms. Its loss is accompanied by installing a more permissive transcriptional landscape that puts cells at a higher risk of additional tumourigenic events at pancreatic islets [18]. Intriguingly, some HIV-1-infected patients develop lipodystrophy syndrome (HALS), characterised by alterations in fatty tissue distribution (lipodystrophy) and systemic metabolic complications. HALS patients typically present a peripheral lipoatrophy of subcutaneous adipose tissue, visceral fat accumulation, lipomatosis, dyslipidemia, and insulin resistance [64]. We wonder if the molecular mechanisms downstream of Atrx loss in vivo include ERV derepression with equivalent consequences on adipose tissue.

We verified that the loss of Atrx does not induce PanNET in the mouse pancreas [34]. Considering the increasingly explored epigenomes and transcriptomes of human PanNETs, based on the expression of aristaless-related homeobox (ARX) and PDX1, the drivers of α and β cell differentiation, respectively, the alterations induced by us in our two β cell-conditional GEMMs (*R.Atrx* and *P.Atrx*) may have been mistargeted. It was described that the most altered endocrine cell population in *ATRX*/*DAXX*-mutated PanNETs harbours a typical landscape signature of α cells (*ARX*^+^/*PDX1*^−^) [38,39]. Therefore, the conditional targeting of β cells may not be ideal. Notwithstanding, we should bear in mind that *Pdx1*-driven genetic alterations target not only β but also α cells, as recently confirmed [35]. This also reinforces the importance of using *Pdx1-Cre* (instead of *Rip-Cre*) to study chromatin remodellers.

The results obtained with our Atrx models interestingly suggest the potential role of ATRX in the homeostasis of human pancreatic endocrine function. T2D is a significant risk factor for PanNET development [65,66], but these patients are also at risk for T2D development [67,68]. An association between metabolic syndrome and GEP-NET patients has also been found [69]. In PanNET patients, the prevalence of longstanding T2D, new-onset T2D, and impaired fasting glucose (IFG) was approximately 12–17%, 8–9%, and 7–26%, respectively [67,68]. Moreover, older PanNET patients (≥60 years) presented longstanding T2D at a higher risk (26%) than the overall population of the same age (below 20%) [67,70]. Specific parameters, such as age and tumour size, are risk factors for T2D in PanNET patients [67]. The prognostic value of preoperative T2D in patients with non-functioning (NF) PanNETs has also been determined: the preoperative new onset of T2D and IFG were associated with aggressive tumour behaviour and poor DFS in patients with NF tumours [67]. A glucose profile assessment is already part of PanNET patient management. As far as we know, no works report a correlation or association between genetic events (such as gene mutations) and HbA1C levels or T2D development. It would be interesting to evaluate the HbA1C levels in PanNET patients presenting the *ATRX* (or *DAXX*) mutation to ascertain its potential involvement in endocrine function and contribution to PanNET disease.

Soon, novel models will likely explore the actual contribution of chromatin remodellers to PanNETs. The new generation of GEMMs could help clarify whether the mutations found in human PanNETs are aetiologically relevant or passenger events [22,24,71,72] and even explore the putative synergistic effects that could arise from multi-targeting approaches. Nevertheless, our works and others have alerted us that tumourigenesis upon ATRX or DAXX loss may probably only occur in human genomes [34,35], and such potential species-specificity should be considered.

## 5. Conclusions

Using the *Pdx1-Cre;Atrx^KO^* GEMM, we reinforced the preponderant role of *Atrx* disruption in the β cells of adult mice with endocrine dysfunction. Unlike *Rip-Cre;Atrx^KO^*, in which a mild compromise of pancreatic homeostasis was perceived in younger ages, *Atrx* disruption in this different driver line had major metabolic implications, leading to obesity and diabetes at young ages. Adiposity led to intrapancreatic fatty infiltration that progressively increased with ageing and resulted in enhanced pancreatic fatty infiltration and peripancratic fat deposits, hampering the interpretation of pancreatic histopathological and hepatic steatosis scores.

## Figures and Tables

**Table 1:** Summary of the contributions of the chromatin remodellers Atrx and Daxx to pancreatic tumourigenesis and inflammageing. ADM, acinar-to-duct cell metaplasia; EF, endocrine fraction; FI, intrapancreatic fatty infiltration; M, Mist1-Cre; P, Pdx1-Cre; PanIN, pancreatic intraepithelial neoplasia; and R, Rip-Cre. Notes: ^a^, All the mutations were analysed at homozygosity, except for our previous study [34]; ^b^, Atrx/Daxx KO as tumourigenic event or accelerator; and ^c^, All studies evaluated male and female mice. Models: ^1^, *Mist1-Cre;Atrx^HOM^*; ^2^, *Mist1-Cre;Kras^HOM^*; ^3^, *Mist1-Cre;Kras^HOM^;Atrx^HOM^*; ^4^, *Pdx1-Cre.Daxx^HOM^*; ^5^, *Pdx1-Cre;Men1^HOM^*; ^6^, *Pdx1-Cre;Men1^HOM^;Daxx^HOM^*; ^7^, *Rip-Cre;Atrx^HOM and HET^*; ^8^, *Pdx1-Cre;Atrx^HOM^*; ^9^, *Pdx1-Cre;Men1^HOM^*; ^10^, *Pdx1-Cre;Men1^HOM^;Atrx^HOM^*; ^11^, *Pdx1-Cre;Pten^HOM^*; ^12^, *Pdx1-Cre;Pten^HOM^;Atrx^HOM^*; ^13^, *Rip-Cre;Men1^HOM^*; ^14^, *Rip-Cre;Men1^HOM^;Daxx^HOM^*; ^15^, *Daxx^HOM^*; and ^16^, *Rip-Cre;Daxx^HOM^*; all the promoters were expressed at heterozygosity.

Model ^a^	Contribution to Tumourigenesis (PDAC/PNET) ^b^	Inflammageing; Islet Size	Sex Differences ^c^	Study
*M.Atrx* ^1^ *M.K* ^2^ *M.K.Atrx* ^3^	Yes (*M.K.Atrx*), as an accelerator in PanIN development (vs. *M.K*)	Yes (*M.Atrx* upon caerulein administration), ADM and fibrosis	Yes (phenotype changes exclusive to females)	[36]
*P.Daxx* ^4^ *P.M* ^5^ *P.M.Daxx* ^6^	No	Yes (*P.M.Daxx* upon caerulein administration), ADM and cystic degeneration; authors attributed to ERVs derepression	No	[18]
*R.Atrx* ^7^	No	Yes, mild inflammageing lesions; abnormal ageing-related EF growth	Yes (endocrine dysfunction is more pronounced in males)	[34]
*P.Atrx* ^8^ *P.M* ^9^ *P.M.Atrx* ^10^	No	No (*P.Atrx* presented the smallest islet size by euthanasia)	No	[35]
*P.Pten* ^11^ *P.P.Daxx* ^12^	No	No	No	
*R.M* ^13^ *R.M.Daxx* ^14^	No	No	No	
*Daxx* ^15^ *R.Daxx* ^16^	No	No	No	
*P.Atrx* ^8^	No	Yes, FI and increased peripancreatic fat deposition	Yes (different onsets of diabetes and obesity)	This study

## Data Availability

Data sharing not applicable.

## Supplementary Materials

The following supporting information can be downloaded at: https://www.mdpi.com/article/10.3390/cancers15113018/s1, Supplementary File S1 (Materials and Methods): Figure S1: Markups of endocrine fraction classifier; Table S1: Primers and genotyping conditions; Table S2: Study population and procedures; Table S3: List of antibodies used in immunohistochemistry; Supplementary File S2 (Results): Figure S2: Pancreatic inflammageing lesions, hepatic steatosis, intrapancreatic fatty infiltration, and macroscopic lesions by three age groups; Figure S3: Normal pancreas; Figure S4: Tumour incidence analysis; Figure S5: Pancreatic tumours; Figure S6: Hemograms; Figure S7: Non-fasted and fasted insulinaemias; Figure S8: GTT-AUC over time; Table S4: Overview of main results by age and genotype; Table S5: Hemograms, all parameters.
