# Supplementary material for: Generation of an Obese Diabetic Mouse Model upon Conditional Atrx Disruption
Source: Cancers (Basel). 2023 Jun 1;15(11):3018. doi: 10.3390/cancers15113018 (PMC10252256; doi:10.3390/cancers15113018)
Supplement: Supplementary file 1 [file cancers-15-03018-s001.zip › cancers-2393979-supplementary.pdf]

Generation of an Obese Diabetic Mouse Model upon Conditional Atrx Disruption

Supplementary Data

Gaspar TB *et al.*, 2023

Table of Contents

Supplementary File S1: Figure S1 – Markups of endocrine fraction classifier. 2

Supplementary File S1: Table S1 – Primers and genotyping conditions 3

Supplementary File S1: Table S2 – Study population and procedures 4

Supplementary File S1: Table S3 – List of antibodies used in immunohistochemistry.......... 5

Supplementary File S2: Figure S2 – Pancreatic inflammageing lesions, hepatic steatosis, intrapancreatic fatty infiltration, and macroscopic lesions by three age groups 6

Supplementary File S2: Figure S3 – Normal pancreas.. 8

Supplementary File S2: Figure S4 – Tumour incidence analysis... 9

Supplementary File S2: Figure S5 – Pancreatic tumours 10

Supplementary File S2: Figure S6 – Hemograms... 11

Supplementary File S2: Figure S7 – Non-fasted and fasted insulinaemias 12

Supplementary File S2: Figure S8 – GTT-AUC over time 13

Supplementary File S2: Table S4 – Overview of main results by age and genotype 14

Supplementary File S2: Table S5 – Hemograms, all parameters 16

Supplementary File S1

Supplementary File S1: Figure S1 – Markups of endocrine fraction classifier.

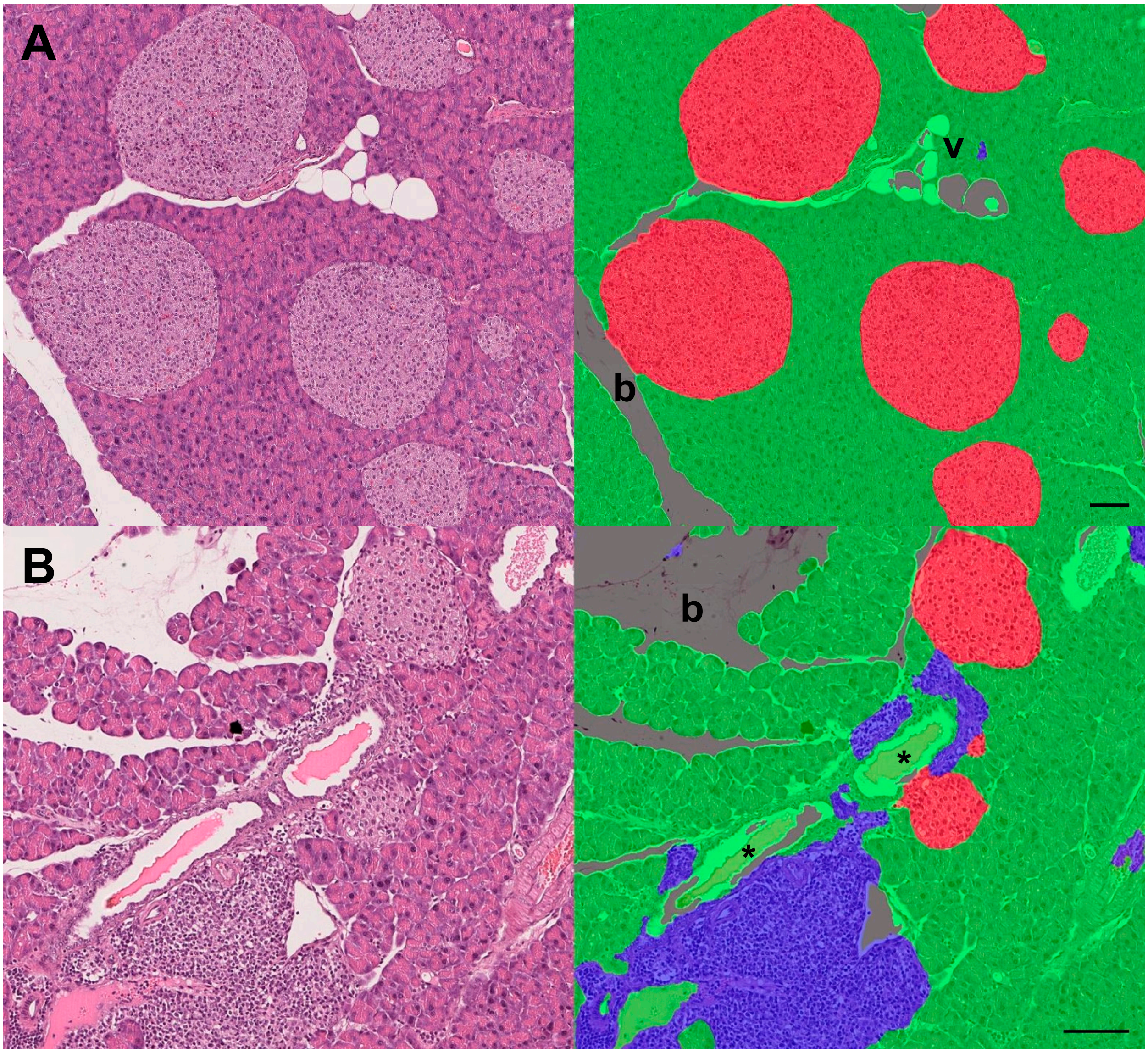

Figure S1 – Markups of endocrine fraction classifier. Markups of the classifier trained in HALO® version 3.5.3577 (Indica Labs) to segment pancreatic islets, exocrine pancreas and inflammatory foci. Our classifier was able to identify islets (red) within the exocrine pancreas (green) (**A**,**B**). The classifier was also trained to identify inflammatory foci (blue) (**C**). Small intrapancreatic vacuoles (v) (**A**) and most of vessel lumens and their content (*) (**B**) were recognized as part of the exocrine tissue. The unstained white areas, that usually comprehend fat tissue, were recognized as 'background' (b) (**A**,**B**).

Supplementary File S1: Table S1 – Primers and genotyping conditions

Table S1 – Primers and genotyping conditions

Primers	Sequence (5'-3')	T_a (º C)	Product length (bp)
**ATRX_int17_F**	GGAGAGGGAAGGAGGAAATG	60	KI = 199<br>WT =150
**ATRX_int17_R**	TAGCCATACCTGCAACCACA	60	
**N10_F**	GTATTGAATTGAAGCACCTTTGTTTGG	55	KI = 370<br>WT = 200
**N12_R**	CTGCCCAAGGCTCCCCCAG	55	
**Cre_F**	ATGTCCAATTTACTGACCGT	55	KI = 100
**Cre_R**	CGCCGCATAACCAGTGAAAC	55	

DNA Extraction: Samples were incubated at 56°C for 2 h on a thermal-shaker with 300 µL of lysis buffer [10 mM Tris (pH 7.5), 400 mM NaCl, 2 mM EDTA (pH 8.0)] (pH 7.3-7.5), 15 µL of 20% sodium dodecyl sulphate (SDS) Cat. No. 428018 (Merck), and 10 µL of 20 mg/mL proteinase K Ref. AM2548 (Ambion RNA by Life Technologies). Then, 100 µL of 6 M NaCl (saturated solution) was added to the extraction mixture, samples were mixed thoroughly by vortexing for 10 s, followed by centrifugation at 14000 rpm for 15 min to precipitate the residual cellular debris. The supernatant was transferred to a clean Eppendorf tube and 800 µL of 100% ethanol was added to each sample, mixed for 10 s, and centrifuged at 14000 rpm for 5 min to pellet the DNA. The DNA pellets were washed with 500 µL of 70% ethanol, followed by centrifugation at 14000 rpm for 5 min. The pellets were completely air dried and suspended in 80 µL of sterile nuclease-free water.

DNA Amplification: For conventional PCR the commercial master mix 2x My Taq HS Mix, Cat. No. Bio-25046 (Bioline) was used. PCR amplifications were carried out in the T-100 Thermal Cycler (Biorad). The reaction mixture was prepared in a 10 µL final volume containing 5 µL of master mix, 1-2 pmol of each primer and 1 µL of genomic DNA (~50-100 ng/µL). All assays included at least one positive control samples and a no-template control (containing all reaction components except the genomic DNA). The amplification protocol included an initial denaturation and enzyme activation at 95°C for 5 min followed by 30 cycles of denaturation at 95°C for 30 s, annealing at 55-60°C (according to each primer) for 90s, extension at 72°C for 30 s and a final extension at 60°C for 10 min.

Supplementary File S1: Table S2 – Study population and procedures

Table S2 – Study population and procedures

		Study population (2018-2022)		
		P.Atrx^{WT}	*P.Atrx*^{HET}	*P.Atrx*^{HOM}
	Euthanasias (n = 218) [a]	57M<br>38 F	61 F	39 M<br>23 F
	HP evaluation of non-tumoural pancreas (n = 136) [b]	36 M<br>22 F	38 F	25 M<br>15 F
	HP evaluation of non-tumoural liver (n = 120) [c]	25 M<br>15 F	36 F	29 M<br>15 F
	IHC and HP characterisation of tumours (n = 22) [d]	9 M<br>9 F	4 F	6 M<br>4 F
	Endocrine fraction (n = 93) [e]	22 M<br>16 F	21 F	21 M<br>13 F
	ELISA (n = 62) [e]	19 M<br>7 F	14 F	18 M<br>4 F
**Longitudinal analyses***	**Weightings** (n = 772) [f]	66 M (n = 181)<br>38 F (n = 128)	61 F (n = 265)	39 M (n = 193)<br>23 F (n = 45)
	**Glycaemias** (n = 207) [f]	22 M (n = 32)<br>19 F (n = 34)	28 F (n = 73)	17 M (n = 51)<br>11 F (n = 17)
	**ipGTTs** (n = 71) [e]	13 M (n = 15)<br>12 F (n = 13)	13 F (n = 15)	9 M (n = 16)<br>9 F (n = 12)
	**Hemograms** (n = 89) [g]	13 M (n = 18)<br>8 F (n = 12)	27 F (n = 36)	8 M (n = 14)<br>9F(n = 9)

P.Atrx^{WT} *Pdx1-Cre*$^{+/-}$*;Atrx*$^{y/wt\ or\ wt/wt}$, ***P.Atrx***^{HET} *Pdx1-Cre*$^{+/-}$*;Atrx*$^{f/wt}$*;* ***P.Atrx***^{HOM} *Pdx1-Cre*$^{+/-}$*;Atrx*$^{y/f\ or\ f/f}$, **F** female mice, **M** male mice. **HEP** humane endpoint, **HP** histopathological, **IHC** immunohistochemistry, **ipGTTs** intraperitoneal glucose tolerance tests. * number of measurements (**n**) are depicted in all genotypes, for both sexes. **Notes: a** euthanasias were either whenever HEP were reached or predetermined; euthanasias occurred at all ages, and were then organised into eight age groups: 3, 6, 9, 12, 15, 18, 21, and 24 mo.; **b** total pancreas slides evaluated with HP score of pancreatic inflammation; **c** total liver slides evaluated with the nonalcoholic fatty liver disease (NAFLD) activity score (NAS); livers with evidence of hyperplasia or discrete benign tumour were also evaluated with NAS; **d** the 29 mice either bear benign or malignant tumours (n = 7 and n = 22, respectively); around 40% of the tumours were characterised with IHC; **e** performed in three age groups: 3, 6, and 12 mo.; **f** assessed at all ages in a longitudinal fashion, and then organised into five age groups (3, 6, 12, 18, and 24 mo.); measurements at T0 of ipGTT were also included; measurements of agitated mice were excluded; 217 and 107 animals were included in weight and glycaemia analyses, respectively; weights of mice by DOD were also included; **b-f** measurements at 9, 15, and 21 mo. were included in 12, 18 and 24 mo. age groups; **g** performed in three age groups: 3, 6, 12 (most animals have more than one measurement over time); ages group of 18 mo. and 24 mo. included in age group of 18 mo.

Supplementary File S1: Table S3 – List of antibodies used in immunohistochemistry

Table S3 – List of antibodies used in immunohistochemistry

Antigen	Species (clonality)	Source	Antibody (Reference)	RRID Code	Antigen retrieval (buffer, minutes)	Dilution (incubation time, hours)	Detection (DAB time, minutes)
**Chromogranin A**	Rabbit (P)	Synaptic Systems	259003	AB_2619972	Steamer (C, 40')	1:400 (1)	2
**Synaptophysin**	Rabbit (M)	Thermo Fisher	9111-S0	AB_149939	Steamer (C, 40')	1:100 (1)	2
**Vimentin**	Rabbit (M)	Cell Signaling	5741	AB_10695459	Steamer (C, 40')	1:250 (ON)	2
**Keratin, Pan**	Mouse (M)	Thermo Fisher	MS-343-P1	AB_61535	Water bath (E, 20')	1:200 (1)	3
**CD45**	Rat (M)	BD Pharmingen	550539	AB_2174426	Microwave (E, 15')	1:10 (ON)	8

M monoclonal, **P** polyclonal, **C** 1x citrate, **E** 1x EDTA, **ON** overnight (16-18 hr), **RTU** ready-to-use

Supplementary File S2

Supplementary File S2: Figure S2 – Pancreatic inflammageing lesions, hepatic steatosis, intrapancreatic fatty infiltration, and macroscopic lesions by three age groups

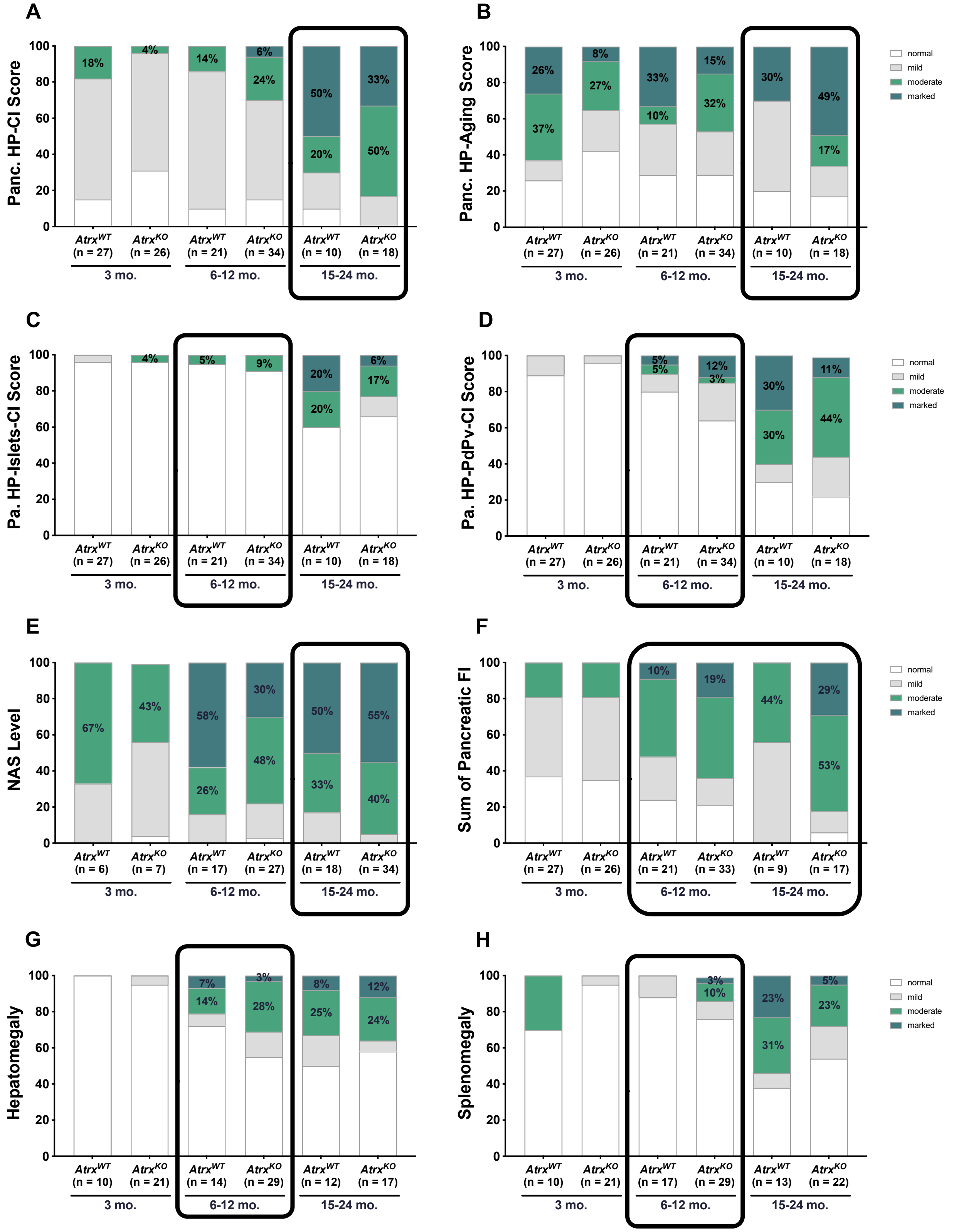

Figure S2 – Pancreatic inflammageing lesions, hepatic steatosis, intrapancreatic fatty infiltration, and macroscopic lesions by three age groups. Distribution by three age groups (3 mo., 6-12 mo., and 15-24 mo.) of eight parameters evaluated with a four-level intensity score: pancreatic histopathological (HP) score of chronic inflammation (CI) was divided in normal (scores 0-1), mild (2-4), moderate (5-7), and marked (≥ 8) (**A**); pancreatic HP score of ageing parameters (that considered ductal/vascular dilation, focal acinary atrophy, and the presence of ductal dysplasia) was divided in normal (scores 0-1), mild (2), moderate (3), and marked (≥ 4) (**B**); pancreatic HP score of CI at istet and at periductal/perivascular (Pd/Pv) locations(**C** and **D**, respectively), and hepatomegaly and splenogaly (**G** and **H**, respectively) were descriminated into 0 (corresponding to absence of the alteration, < 5%), 1 (low-grade lesion, 5-33% altered), 2 (moderate-grade lesion, 33-66%), and 3 (high-grade lesion, > 66% altered); NAS level was divided in normal (scores 0-2), mild (3-4), moderate (5-7), and marked (≥ 8) (**E**). The stratification of intrapancreatic fatty infiltration (FI) summed with the extent of peripancreatic fat which may include pancreatic fatty replacement (FR) was the following: normal (scores 0-1), mild (2), moderate (3-4), and marked (5) (**F**). The black rectangles call the attention for the comparisons in which *P.Atrx*^{KO} present increased levels than age-matched controls.

Supplementary File S2: Figure S3 – Normal pancreas

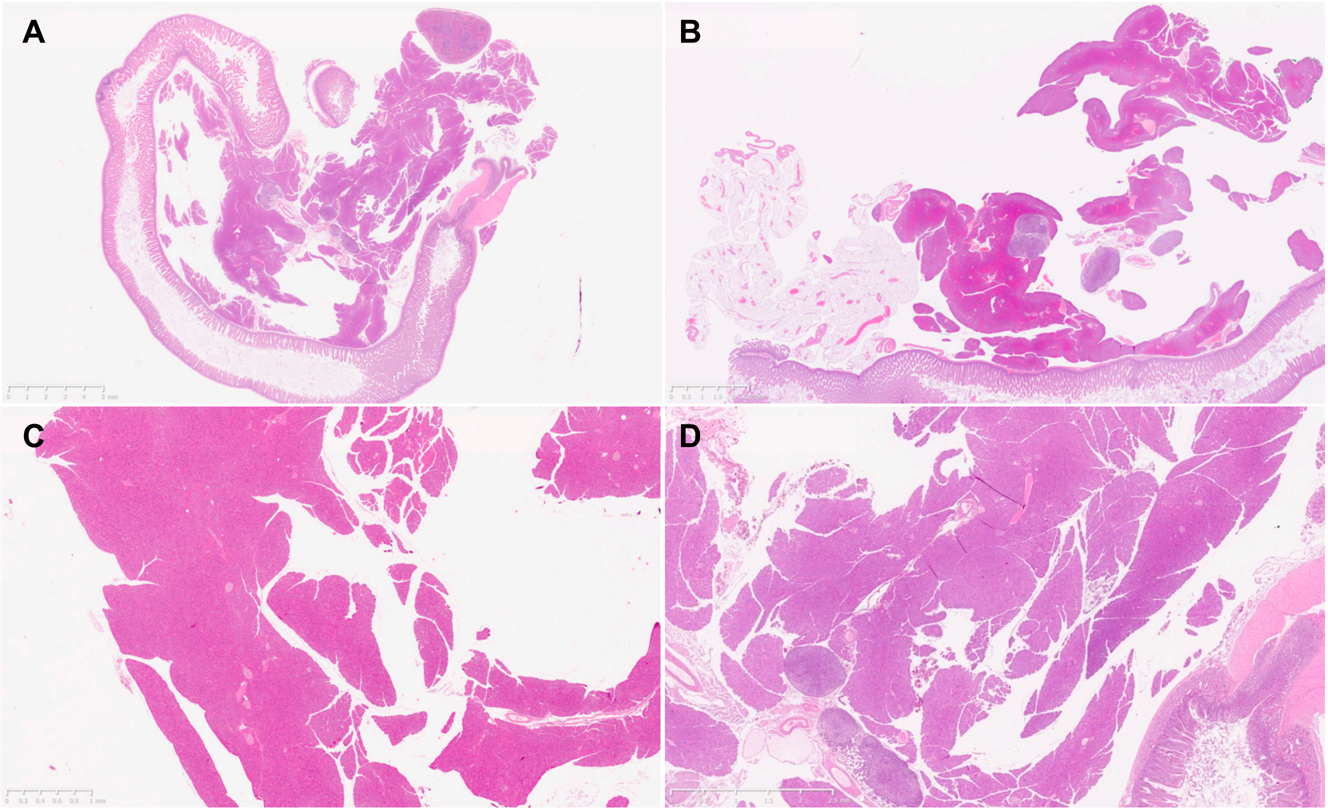

Figure S3 – Normal pancreas. Representative images of normal pancreas not infiltrated with fat (A,C,D), attributed a score 0 of intrapancreatic fatty infiltration; peripancreatic fat tissue can be observed in image (B), attributed a score 2 concerning this parameter.

Supplementary File S2: Figure S4 – Tumour incidence analysis

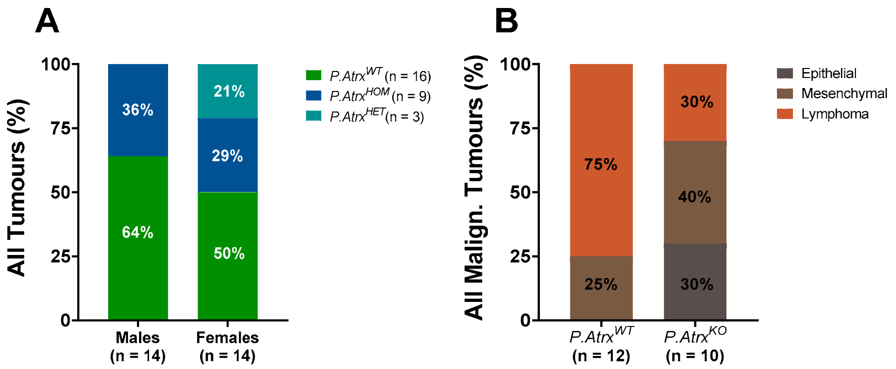

Figure S4 – Tumour incidence analysis. All tumours distribution by genotypes in males and females mice (**A**) and distribution of all malignant tumours by three phenotypes (epithelial, mesenchymal, and lymphoma) in *P.Atrx*^{WT} and *P.Atrx*^{KO} mice **(B)**.

Supplementary File S2: Figure S5 – Pancreatic tumours

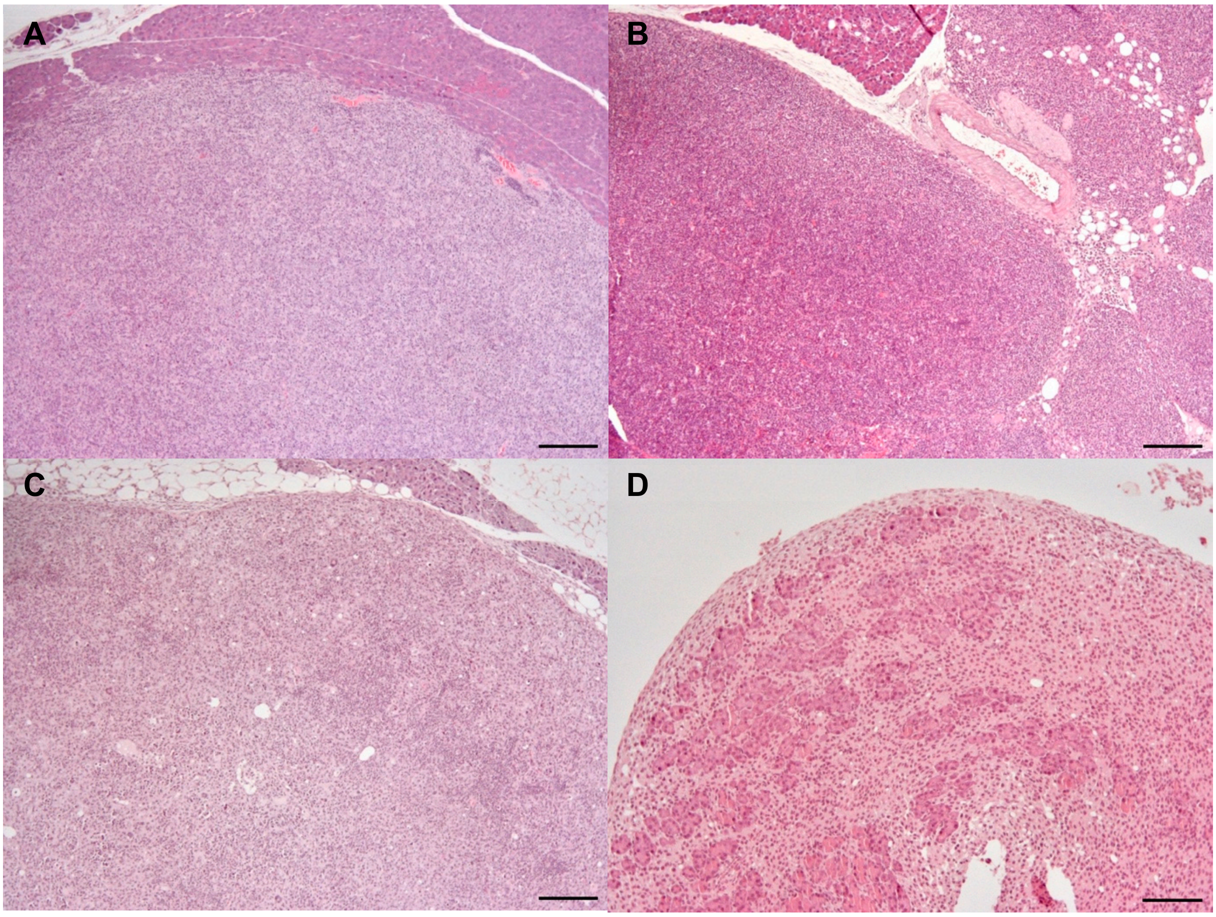

Figure S5 – Pancreatic tumours. Representative images of the four pancreatic tumours. Morphology was compatible with lymphoma (A,B) and mesenchymal tumours (C,D). Scale bar was set at 4 µm (A–C) and 10 µm.

Supplementary File S2: Figure S6 – Hemograms

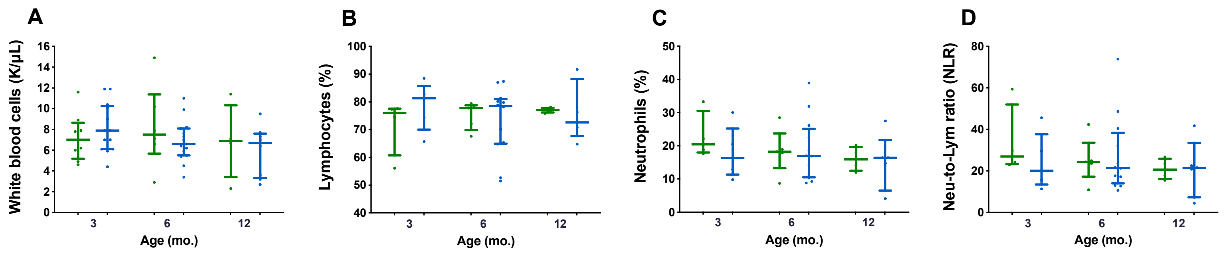

Figure S6 – Hemograms. Distribution by three age groups (3, 6, and 12 mo.) of hemograms. The hemogram analysis ensures that mice did not develop any systemic inflammatory state. The values of white blood cell count (**A**), lymphocytes percentage (**B**), neutrophile percentage (**C**), and neutrofile to lymphocyte ratio (**NLR**) do not differ between genotype groups. **Green** *P.Atrx*^{WT}, **blue** *Atrx*^{KO}. Data is shown as median +/- IQR.

Supplementary File S2: Figure S7 – Non-fasted and fasted insulinaemias

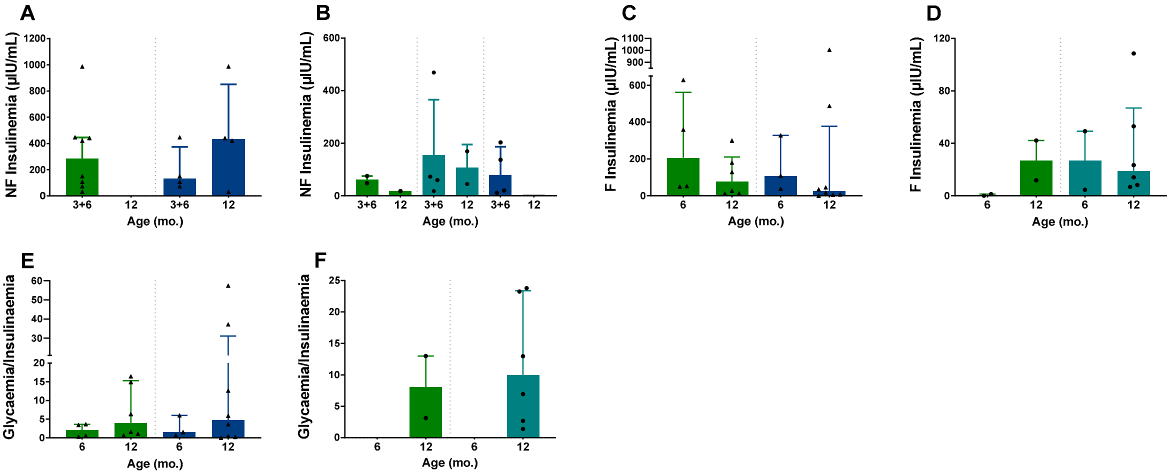

Figure S7 – Non-fasted and fasted insulinaemias. Non-fasted (NF) insulinaemia values were obtained from frozen serum collected by the time of euthanasia of male **(A)** and female **(B)** mice. Insulin of NF *P.Atrx*^{HOM} male mice by 12 mo. is higher than age-matched controls. Insulin of NF *P.Atrx*^{HET} females is higher than age-matched controls at all ages; NF insulinaemia seems to increase with aging specifically in male *P.Atrx*^{HOM} mice. But NF insulinaemia seems to drecrease in females of all genotypes with aging. Fasted (F) insulinaemia values were obtained from 6-hour fasted male **(C)** and female **(D)** mice. *Atrx*^{HOM} male mice exhibit decreased F insulinaemia than age-matched controls; median insulinaemia values seem to decrease in males of all genotypes with aging. *Atrx*^{HET} female mice exhibit higher F insulinaemia than controls by 6 mo.; The 6-hour fasted glycaemia/insulinaemia ratios also indicate that *P.Atrx*^{KO} have a tendency for higher values **(E,F)**. **Green** *P.Atrx*^{WT}, **tail** *P.Atrx*^{HET}, **ocean** blue *P.Atrx*^{HOM}. Triangles and circles represent values of male and female mice, respectively. All values are represented as median +/- IQR.

Supplementary File S2: Figure S8 – GTT-AUC over time

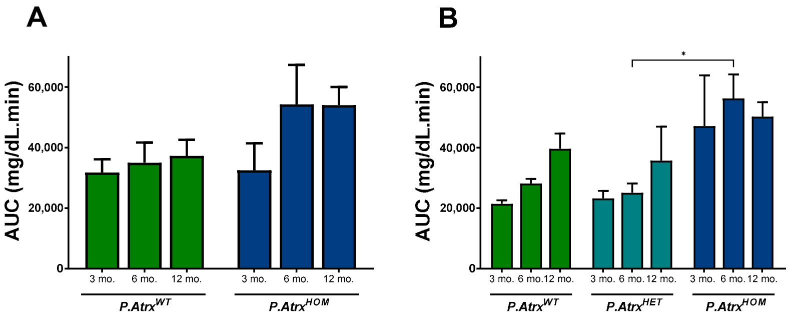

Figure S8 – GTT-AUC over time. Distribution by three age groups (3, 6, and 12 mo.) of GTT-AUC. Plotting the values this way allowed the identification the increasing GTT-AUC profile over time in all genotype groups **(A,B)**, especially in *P.Atrx*^{WT} females **(B)**. * $p < 0.05$. **Green** *P.Atrx*^{WT}, **tail** *P.Atrx*^{HET}, **blue** *Atrx*^{HOM}. Data is shown as mean +/- SD.

Supplementary File S2: Table S4 – Overview of main results by age and genotype

Table S4 – Overview of main results by age and genotype

		3 mo.				6 mo.				12 mo.				18 mo.				24 mo.				
		x̄/M	SD/IQR	n	P	x̄/M	SD/IQR	n	P	x̄/M	SD/IQR	n	P	x̄/M	SD/IQR	n	P	x̄/M	SD/IQR	n	P	total n
Weights	M and F P.Atrx^{WT}	25.93	5.04	73	a, e	31.53	6.70	59	a, f, i, #	36.18	9.26	91	f, k, #, ✳, π	35.22	11.69	84	m	38.20	13.20	26		333
	M and F P.Atrx^{HOM}	29.42	7.19	53	b, e	34.96	8.39	57	b, g, i	43.54	10.68	89	g, k, π	44.48	14.89	75	l, m, #,✳, Ω	35.74	13.16	31	l	305
	F P.Atrx^{HOM}	21.14	3.49	12	c	29.81	7.52	15	c	36.38	12.74	8		42.80	15.93	12		30.55	1.06	2		49
	F P.Atrx^{HET}	22.25	3.97	59	d	28.36	4.85	51	d, h	34.86	6.77	106	h, j	38.71	10.96	76	j	34.76	12.83	33		325
	Correlations	-				-				-				-				-				**963**
Glycaemia	M and F P.Atrx^{WT}	140.88	15.21	16	a, b, #	168.08	26.74	13	a, d, #, ✳	173.10	33.09	31	e, #, Ω	160.20	43.69	10		125.00	29.70	2		72
	M and F P.Atrx^{HOM}	229.33	87.27	15	b, e, #	266.81	97.49	16	c, d	208.32	50.01	28	c, e	171.44	46.82	9	#	206.25	188.94	4		72
	F P.Atrx^{HOM}	299.50	153.46	4		314.00	150.25	5		187.33	66.91	3		168.50	72.95	4		488.00	0.00	1		17
	F P.Atrx^{HET}	148.53	20.06	15		151.57	23.55	21		158.82	33.14	28		157.86	33.37	7		145.40	14.64	5		76
	Correlations	-				# Pearson's r = 0.718 ▲▲				# Pearson's r = 0.397 ▲				# Pearson's r = -0.757 ▼▼				-				**220**
GTT-AUC	M and F P.Atrx^{WT}	25474.00	10317.00	8	b, #	30368.00	11783.00	9	d, e, ✳	37290.00	8317.00	11	d, g, ✳									28
	M and F P.Atrx^{HOM}	36173.00	21060.00	9	a, b, #	53100.00	19073.00	9	a, e	55448.00	9165.00	9	g									27
	F P.Atrx^{HOM}	47029.00	31731.00	4	c	53100.00	15109.00	5	f	52388.00	n.a.	2										11
	F P.Atrx^{HET}	22372.50	4461.00	4	c	26648.00	5824.00	5	f	33075.50	19896.00	6										15
	Correlations	# Kendall's τ = 0.571 ▲▲ (WT)<br># Kendall's τ = 0.778 ▲▲ (HOM)				✳ Kendall's τ = 0.667 ▲				✳ Kendall's τ = 0.477▲												**70**
EF	M and F P.Atrx^{WT}	0.63	0.75	13	✳	0.97	0.92	8		1.30	1.70	10	Ω	1.27	n.a.	3		1.71	1.19	4		38
	M and F P.Atrx^{HOM}	0.36	0.30	7	a, ✳	0.89	0.85	6	a	1.18	1.81	10		1.48	0.33	7		2.40	n.a.	2		32
	F P.Atrx^{HOM}	0.36	n.a.	3		1.27	n.a.	3		0.99	n.a.	3		1.20	n.a.	3		n.a.	n.a.	0		12
	F P.Atrx^{HET}	0.55	0.22	4		0.76	0.43	4	b	1.35	0.50	7	b	1.45	n.a.	2		1.45	n.a.	3		20
	Correlations	-				-				Ω Kendall's τ = 0.905 ▲▲▲								-				**90**
HP-CI-Pa	M and F P.Atrx^{WT}	3.00	2.00	27	a	4.00	1.00	11		3.00	3.00	10		8.00	12.00	5		6.00	6.00	5		58
	M and F P.Atrx^{HOM}	1.50	3.00	10	a	3.00	2.00	8		4.00	4.00	12		7.00	5.00	7	✳	7.00	n.a.	3		40
	F P.Atrx^{HOM}	0.50	4.00	4		3.00	n.a.	3		3.00	4.00	4	b	7.00	n.a.	3	b	12.00	n.a.	1		15
	F P.Atrx^{HET}	2.50	2.00	16		2.00	4.00	6		5.00	6.00	7		7.00	5.00	5		7.50	2.00	4		38
	Correlations	-				-				-				✳ Kendall's τ = 0.651 ▲				-				**136**
Sum of pancreatic FI	M and F P.Atrx^{WT}	2.00	1.00	27	a, ✳	3.00	2.00	11	a, c	2.00	2.00	10	c, d	3.00	2.00	4		2.00	2.00	5		57
	M and F P.Atrx^{HOM}	2.00	1.00	10	✳	3.50	3.00	8		3.50	1.00	12	d	5.00	2.00	7		3.00	n.a.	3		40
	F P.Atrx^{HOM}	2.00	1.00	4	b	4.00	n.a.	3	b	3.50	2.00	4		5.00	0.00	3	e	2.00	n.a.	1		15
	F P.Atrx^{HET}	2.00	2.00	16		1.50	3.00	4		3.00	3.00	7		3.00	2.00	5	e	4.00	2.00	4		36
	Correlations	✳ Kendall's τ = 0.467 ▲ (WT)<br>✳ Kendall's τ = 0.900 ▲▲▲ (HOM)				-				-				-				-				**133**
HP-NAS-Li	M and F P.Atrx^{WT}	4.50	1.00	6	a	7.00	3.00	11	a, b	8.00	2.00	11	b, π	8.00	6.00	7		7.00	6.00	5		40
	M and F P.Atrx^{HOM}	4.00	3.00	9		5.50	5.00	10		7.00	4.00	12	π	7.00	4.00	10	Ω	9.00	n.a.	3		44
	F P.Atrx^{HOM}	4.50	3.00	4		4.00	n.a.	3		6.50	3.00	4		12.00	n.a.	3		6.00	n.a.	1		15
	F P.Atrx^{HET}	4.50	2.00	14		4.50	3.00	6		6.00	3.00	7		8.00	6.00	6		9.00	n.a.	3		36
	Correlations	-				-				π Kendall's τ = 0.584 ▲ (WT)<br>π Kendall's τ = 0.520 ▲ (HOM)				Ω Kendall's τ = 0.569 ▲				-				**120**

Overview of the results of eight parameters distributed by **five age groups** (3, 6, 12, 18, and 24 months (mo.)) and by **four genotype groups** (male and female *P.Atrx*^{WT}, male and female *P.Atrx*^{HOM}, female *Atrx*^{HOM}, and female *P.Atrx*^{HET}). For each parameter, statistical comparisons were performed to assess **age-related changes** – over time (3 vs. 6 mo., 6 vs. 12 mo., 12 vs. 18 mo., 18 vs. 24 mo.), for each genotype group – and to assess **genotype-related changes** – within each age group, among genotypes (*Atrx*^{WT} *vs Atrx*^{HOM} of both sexes, and *Atrx*^{HET} vs. *Atrx*^{HOM} females). Correlations were assessed all pairs of parameters, between *P.Atrx*^{WT} *vs. P.Atrx*^{HOM} of both sexes; **#/✱** indicate correlation pairs (read vertically), **triangles** indicate correlation strength and direction. Data is presented as mean (**x̄**) and standard deviation (**SD**) (weights and glycaemias) or median (**M**) and interquartile range (**IQR**) (all the remaining parameters), count (**n**) and *p*-value (**P**); a red-yellow-green colour scheme was used based on the mean/median values (red represent the highest value); all *p*-values < 0.05 are represented by pairs of lowercase letters – **same letter** represent statistically significant difference within each parameter. Independent t-tests and Pearson's correlation was performed in larger groups (n ≥ 15), while Mann-Whitney tests and Kendall's nonparametric tests were performed in smaller groups (n < 15). **GTT-AUC** glucose tolerance test's area under the curve, **EF** endocrine fraction, **HP-CI-Pa** histopathological evaluation of chronic inflammation (CI) in pancreas slides (Pa), **FI** fatty infiltration, **P-NAS-Li** histopathological evaluation of non-alcoholic fatty liver disease activity score (NAS) in liver slides (Li). Sum of pancreatic FI includes intrapancreatic fatty infiltration and the extent of peripancreatic fat which may include pancreatic fatty replacement.

Supplementary File S2: Table S5 – Hemograms, all parameters

Table S5 – Hemograms, all parameters

	3			6			12		
**Values**	***P.Atrx*** ^{KO}	***P.Atrx*** ^{WT}	**Total**	***P.Atrx*** ^{KO}	***P.Atrx*** ^{WT}	**Total**	***P.Atrx*** ^{KO}	***P.Atrx*** ^{WT}	**Total**
Average of RBC	8.6	8.5	8.6	8.7	9.1	8.8	8.8	9.1	8.9
StdDev of RBC	1.1	1.4	1.2	1.3	0.9	1.2	0.7	0.9	0.7
Count of RBC	***15***	***10***	***25***	***15***	***6***	***21***	***13***	***6***	***19***
Min. of RBC	6.0	5.6	5.6	5.5	7.3	5.5	7.4	8.0	7.4
Max. of RBC	10.1	10.6	10.6	10.2	9.8	10.2	10.1	10.2	10.2
Average of % Ret	4.5	4.4	4.5	4.2	4.5	4.3	5.0	4.5	4.9
StdDev of % Ret	3.0	1.9	2.5	1.4	1.1	1.3	1.3	1.0	1.2
Count of % Ret	***11***	***8***	***19***	***10***	***3***	***13***	***9***	***5***	***14***
Average of Ret	332.2	339.6	335.1	352.4	392.8	362.5	440.4	395.9	424.5
StdDev of Ret	183.9	96.8	151.8	101.5	138.3	106.3	130.8	83.2	114.6
Count of Ret	***12***	***8***	***20***	***9***	***3***	***12***	***9***	***5***	***14***
Average of WBC	8.1	7.2	7.8	6.9	8.3	7.3	5.9	6.9	6.2
StdDev of WBC	2.4	2.3	2.3	2.0	4.0	2.7	2.5	3.7	2.8
Count of WBC	***13***	***8***	***21***	***15***	***6***	***21***	***8***	***4***	***12***
Min. of WBC	4.4	4.6	4.4	3.4	2.9	2.9	2.7	2.3	2.3
Max. of WBC	11.9	11.6	11.9	11.0	14.9	14.9	9.5	11.4	11.4
Average of % Neu	17.9	23.0	20.2	18.8	18.4	18.7	15.3	16.0	15.6
StdDev of % Neu	7.8	7.1	7.6	9.5	7.0	8.6	8.1	3.7	6.6
Count of % Neu	***5***	***4***	***9***	***12***	***5***	***17***	***7***	***4***	***11***
Min. of % Neu	9.8	17.7	9.8	8.8	8.7	8.7	4.1	12.0	4.1
Max. of % Neu	30.0	33.3	33.3	38.9	28.5	38.9	27.5	20.2	27.5
Average of % Lym	78.5	71.4	75.4	73.1	75.0	73.7	76.0	77.0	76.4
StdDev of % Lym	8.8	10.3	9.6	12.2	5.0	10.5	10.2	0.9	8.0
Count of % Lym	***5***	***4***	***9***	***12***	***5***	***17***	***7***	***4***	***11***
Min. of % Lym	65.7	56.1	56.1	51.5	67.6	51.5	64.8	75.9	64.8
Max. of % Lym	88.5	77.6	88.5	87.4	79.4	87.4	91.7	78.1	91.7
Average of % Mon	1.9	4.4	3.0	7.2	4.9	6.5	6.9	5.5	6.4
Average of Neu	1.6	2.0	1.7	1.3	1.3	1.3	0.8	1.1	0.9
StdDev of Neu	0.5	0.5	0.5	0.7	1.0	0.8	0.4	0.5	0.4
Count of Neu	***5***	***4***	***9***	***12***	***5***	***17***	***7***	***4***	***11***
Average of Lym	7.4	6.3	6.9	4.9	5.2	5.0	4.4	5.4	4.8
StdDev of Lym	3.1	2.2	2.7	1.7	1.8	1.7	2.2	2.9	2.4
Count of Lym	***5***	***4***	***9***	***12***	***5***	***17***	***7***	***4***	***11***
Average of PLT	602.8	566.5	588.3	651.4	529.3	620.9	662.6	672.3	666.1
StdDev of PLTs	143.9	324.6	192.6	141.1	320.2	190.2	102.8	34.9	82.0
Count of PLTs	***3***	***2***	***5***	***9***	***3***	***12***	***7***	***4***	***11***
Average of NLR	24.4	34.1	28.7	27.7	25.2	26.9	21.7	20.9	21.4
StdDev of NLR	13.6	17.2	15.1	18.6	11.2	16.4	13.2	5.1	10.6
Count of NLR	***5***	***4***	***9***	***12***	***5***	***17***	***7***	***4***	***11***
Average of PLR2	10689.4	11398.0	10972.8	15914.4	11494.6	14809.5	19542.9	18054.4	19001.6
StdDev of PLR	7974.4	2305.0	5768.4	5188.4	3500.3	5079.5	11639.9	14775.0	12138.7
Count of PLR	***3***	***2***	***5***	***9***	***3***	***12***	***7***	***4***	***11***
Average of SII	120.5	196.5	150.9	203.4	96.7	176.7	144.6	141.6	143.5
StdDev of SII	60.9	5.2	60.0	132.6	70.9	126.6	85.8	41.2	70.2
Count of SII	3.0	2.0	5.0	9.0	3.0	12.0	7.0	4.0	11.0

RBC red blood cells, **Ret** reticulocytes, **WBC** white blood cells, **Neu** neutriophils, **Lym** lymphocytes, **PLTs** platelets, **NLR** neutrophil-to-lymphocyte ratio. **PLR** platelet-to-lymphocyte ratio, **SII** systemic immune-inflammation index ((PLTs x Neu)/Lym).
